# Chronic Pain: Epidemiology, Pathophysiology, and Clinical Management

**DOI:** 10.1002/mco2.70756

**Published:** 2026-05-06

**Authors:** Zhihao Shang, Zijiao Tian, Zhaoquan Wang, Zerui Yang, Ziyao Wang, Zikang Wang, Ziqing Wang, Xingyuan He, Yu Yan, Yuanfeng Gong, Siyuan Xu, Xinyi Li, Guihua Tian

**Affiliations:** ^1^ Beijing Institute of Integrated Traditional Chinese and Western Medicine Beijing Friendship Hospital Capital Medical University Beijing China; ^2^ Dongzhimen Hospital Beijing University of Chinese Medicine Beijing China; ^3^ Beijing Friendship Hospital Capital Medical University Beijing China; ^4^ Danyang Hospital of Traditional Chinese Medicine Teaching Hospital of Nanjing University of Chinese Medicine Danyang China

**Keywords:** chronic pain, global burden, maladaptive neural plasticity, multidisciplinary approaches, neuro–immune interactions, precision medicine

## Abstract

Chronic pain represents a pervasive global health crisis and a leading cause of disability, yet its management remains challenged by the intricate heterogeneity of its underlying mechanisms. Transcending traditional symptom‐based paradigms, chronic pain is now recognized as a distinct disease entity driven by multidimensional maladaptive plasticity. In this review, we synthesize the current landscape of chronic pain, bridging macrolevel epidemiological burdens with microlevel pathophysiological insights. We dissect the complex biological networks driving pain chronification, ranging from peripheral sensitization and ion channel dysfunction to central synaptic reorganization, spinal disinhibition, and maladaptive neuro–immune crosstalk involving glial activation and autoantibody‐mediated mechanisms. Notably, we highlight emerging frontiers, including sexual dimorphism in immune signaling, metabolic reprogramming, and epigenetic memory. Furthermore, we critically evaluate the evolution of clinical management strategies, integrating pharmacological innovations, advanced neuromodulation, and digital therapeutics. Finally, we address the persistent translational chasm between basic discovery and clinical efficacy, advocating for a paradigm shift from “one‐size‐fits‐all” approaches toward mechanism‐based precision medicine—underpinned by robust biomarkers and deep phenotyping—to revolutionize therapeutic outcomes.

## Graphical abstract

The multidimensional nature of chronic pain is illustrated through four interconnected domains. *(Left) Epidemiology and Global Burden*: Chronic pain affects approximately one in five individuals globally, contributing to a rising trend in disability‐adjusted life years (YLDs) and a significant socioeconomic impact. *(Middle‐Left) Pathophysiology Inputs*: Peripheral sensitization is driven by diverse etiologies, including nerve injury, bone cancer‐induced nociception, and the release of an “inflammatory soup” (a cocktail of cytokines, protons, and lipids) at the site of tissue damage. *(Middle‐Right) CNS Processing*: Chronic pain is maintained through central sensitization, where spinal and cortical neurons exhibit heightened excitability. This process is further amplified by neuro–glia crosstalk, involving the activation of microglia and astrocytes that modulate synaptic transmission. *(Right) Clinical Management*: A multimodal therapeutic approach is required, integrating traditional pharmacotherapy, neuromodulatory techniques such as acupuncture, and emerging digital health technologies (wearable monitors and mobile health apps) to achieve personalized pain relief and functional recovery.

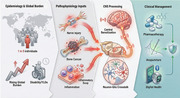

## 1 Introduction

Chronic pain represents one of the most prevalent and burdensome global health challenges, yet it has historically been marginalized as a mere epiphenomenon of other conditions [1]. Accumulating evidence from neuroscience and epidemiology has dismantled this reductive view, leading the International Association for the Study of Pain (IASP) and the World Health Organization (WHO) to classify chronic pain as a distinct disease entity in the International Classification of Diseases, 11th Revision [2]. In line with IASP's contemporary terminology, chronic pain is further stratified into three core subtypes: nociceptive pain, which arises from tissue injury or inflammation that activates nociceptors; neuropathic pain, which stems from lesions or dysfunction of the somatosensory nervous system; and nociplastic pain, which is defined as pain resulting from maladaptive functional remodeling of central pain processing pathways in the absence of clear tissue or nerve damage. This classification underscores that chronic pain is a persistent pathological state driven by multidimensional neural, physiological, and psychological aberrations, rather than a passive sequela of a single peripheral lesion [3, 4, 5]. Transcending archaic linear “stimulus‐response” models, the Gate Control Theory and subsequent advances in central plasticity have anchored the contemporary conceptual framework of pain as a disorder of malleable neural networks, providing the theoretical basis for understanding its intractability and self‐sustaining nature [6].

Within this framework, basic research and clinical practice are shifting from a solely biomedical perspective toward a biopsychosocial (BPS) model [7], deeply integrating precision medicine, neuroimaging, multiomics, and digital health tools. Extensive research has revealed the substantial biological heterogeneity underlying chronic pain. While aberrant ion channel function and axonal regeneration in peripheral nociceptors, combined with synaptic plasticity changes and disinhibition in the spinal cord and brain, collectively shape a persistent “hypersensitive” network [8], this is compounded by glia‐driven neuro–immune crosstalk [9], endocrine–circadian rhythm imbalances [10], and maintenance loops involving epigenetic and metabolic reprogramming [11] facilitate the transition from an acute injury to a long‐term systemic disorder.

Concurrently, the landscape of chronic pain research is evolving rapidly, particularly regarding pathophysiological mechanisms and emerging therapeutic targets, with recent basic and clinical studies significantly outpacing the scope of existing reviews [12]. Current literature often focuses on single pain modalities, such as neuropathic pain [13], fibromyalgia, or chronic low back pain (CLBP) [14], rarely integrating the epidemiological landscape, multiscale pathological mechanisms, and clinical management strategies within a single comprehensive synthesis. Therefore, it is timely to construct a unified framework spanning from population burden to molecular mechanisms and intervention strategies, serving as a reference for both clinical decision‐making and fundamental research. This review is structured to provide such a synthesis. We begin with a macroscopic epidemiological perspective, integrating the latest global data to reveal the severity and ubiquity of chronic pain as a public health imperative [15]. Subsequently, we delve into the microscopic level to systematically dissect the complex pathophysiology central to understanding the intractability and heterogeneity of pain. Here, we highlight frontier mechanisms maintaining the chronic pain state, including neuro–immune interactions, glial activation, and epigenetic modifications. Building on these mechanistic insights, we then comprehensively evaluate existing and cutting‐edge management strategies, summarizing progress in preclinical and clinical trials of emerging therapies, and emphasizing the paradigm shift from a “one‐size‐fits‐all” approach to mechanism‐based, personalized precision medicine. Finally, we conclude by synthesizing key insights and offering perspectives on future directions for research and clinical translation.

## 2 Epidemiology of Chronic Pain

Chronic pain constitutes a global public health challenge with profound implications [16]. Recent epidemiological analyses indicate that approximately 20–30% of adults are affected, with up to one‐third of the population potentially experiencing persistent pain at any given point [17]. This burden is pervasive, transcending geographical and demographic boundaries, and ranks among the leading causes of disability, a trend particularly pronounced in ageing societies. This section delineates the global prevalence from a public health perspective, comparing regional disparities, defining risk factors in susceptible populations, and examining the associated socioeconomic costs to underscore the clinical significance of chronic pain.

### 2.1 Global Prevalence and Distribution

Epidemiological studies demonstrate a high prevalence of chronic pain worldwide; globally, on average, one in five individuals is affected [16, 18]. For certain specific subtypes, prevalence rates can reach as high as 47% globally [19]. While such variability is partly attributable to differences in definitions and measurement methodologies, the ubiquity of the condition across nations and regions is undeniable. In the United States, for instance, an estimated 20.9% of adults (approximately 51.6 million individuals) experienced chronic pain between 2019 and 2021 [20]; recent survey data from 2023 suggest this proportion has risen to approximately 24.3% [21]. Although specific prevalence rates may vary across countries and regions due to demographic structures, cultural contexts, and diagnostic criteria, the universal footprint of chronic pain remains a critical global concern.

The global distribution of chronic pain exhibits distinct patterns. Generally, high‐income countries, characterized by more advanced population ageing, tend to report higher age‐standardized prevalence rates. Analysis based on the Global Burden of Disease (GBD) study indicates that the age‐standardized prevalence of chronic pain is highest in regions with a high Socio‐Demographic Index [22]. This suggests that in developed regions, greater longevity and a higher proportion of elderly individuals contribute to the increased frequency of chronic pain. Conversely, while overall prevalence may appear marginally lower in some developing nations, the heavy reliance on manual labor and constraints in healthcare resources mean the condition still imposes a substantial burden on individual health and societal development. Notably, cultural context and socioeconomic status also influence the reporting and perception of chronic pain; a study spanning 52 countries revealed significant cross‐national variations in prevalence, reflecting the potential impact of national‐level social, economic, and political factors on pain behavior [23].

Within the spectrum of common chronic pain conditions, CLBP is the most prevalent and strongly associated with disability. Based on WHO and GBD estimates, the point prevalence was approximately 7.5% (∼577 million cases) in 2017, rising to ∼619 million in 2020, with projections suggesting a surge to 843 million by 2050 [24, 25]. While higher rates in high‐income regions are driven by ageing populations and lifestyle factors, manual laborers in developing regions are also significantly susceptible [26]; prevalence typically escalates with age, reaching high levels in older cohorts [27]. Osteoarthritis (OA) constitutes the primary source of chronic joint pain, affecting approximately 528 million patients globally in 2019—a 113% increase since 1990, representing roughly 7% of the global population [28]; knee OA is the most pervasive form (∼595 million), predominantly affecting individuals over 40 years of age, followed by hip and hand involvement [29, 30]. Fibromyalgia, characterized by widespread musculoskeletal pain, fatigue, and sleep disturbances, has an estimated prevalence of 1.78% (95% CI 1.65–1.92%) in the general population, reaching ∼3.98% in women while remaining significantly lower in men [31]; this pronounced female preponderance is a consistent epidemiological finding across multiple studies [32]. Neuropathic pain, arising from lesions or diseases of the somatosensory system (such as diabetic peripheral neuropathy and postherpetic neuralgia), affects approximately 7–10% of the general population [33], with rates escalating in high‐risk groups [34]; clinically, it presents with burning or electric‐shock‐like pain and dysesthesia, posing significant therapeutic challenges [35]. Chronic headache imposes a similarly heavy burden: the prevalence of migraine in the adult population is approximately 18.1% [36]; tension‐type headache is even more ubiquitous (26.1% attack rate), frequently undergoing chronification and affecting a substantial proportion of adolescents and young adults annually [37, 38]; indeed, GBD analyses have historically ranked migraine and CLBP as leading causes of years lived with disability [39]. Regarding cancer‐related pain, recent systematic reviews and meta‐analyses estimate an overall prevalence of approximately 44.5%, with ∼30.6% classified as moderate‐to‐severe [40]. Stratification by disease stage reveals pain in ∼39.3% of patients following curative treatment, 55.0% during anticancer therapy, and 66.4% in those with advanced, metastatic, or terminal disease, of whom ∼38% report moderate‐to‐severe intensity [41]. Data from the US NHIS further suggest that ∼34.6% of cancer survivors experience chronic pain, with 16.1% suffering from high‐impact chronic pain—equating to approximately 5.39 million individuals [42]. Collectively, these findings underscore that cancer pain persists throughout the clinical trajectory, intensifying with disease progression and acting as a critical determinant limiting quality of life (QoL) and functional recovery in oncology patients.

In summary, chronic pain exhibits a high global prevalence; although regional and demographic variations exist, its impact on public health is universal. Common subtypes—including low back pain, arthritic pain, headache, fibromyalgia, and neuropathic pain—collectively constitute the majority of the chronic pain burden, each with distinct demographic predilections and epidemiological characteristics (Table 1). These data delineate the global landscape of chronic pain and underscore the imperative for targeted prevention and intervention strategies tailored to specific pain modalities.

**TABLE 1:** Global burden and demographic profile of major chronic pain conditions.

Pain type	Representative conditions/sites	Global burden (cases/prevalence)	Key demographic characteristics	References
Chronic low back pain (CLBP)	Chronic nonspecific lumbar/low back pain	Point prevalence 7.5% in 2017 (577 million individuals); ∼619 million in 2020; projected to reach ∼843 million by 2050	Prevalence increases with age, peaking in older cohorts; rates are higher in high‐income countries due to ageing and sedentary lifestyles, yet manual laborers in developing regions are also significantly affected	[24, 25, 26, 27]
Osteoarthritis (OA)	Degenerative arthritis of the knee, hip, and small joints	528 million individuals affected globally in 2019 (7% of the global population); knee OA is the most common form, affecting ∼595 million	Predominantly affects individuals aged ≥40 years, rising markedly with age; high‐risk groups include those with excess weight, occupations involving joint loading, and postmenopausal women	[28, 29, 30]
Fibromyalgia	Widespread musculoskeletal pain, tender points	Prevalence in the general population is ∼1.78% (95% CI 1.65–1.92); ∼3.98% in women, significantly lower in men	Most common in middle‐aged women, frequently comorbid with fatigue, sleep disturbances, and depression/anxiety; typically follows a chronic, fluctuating course	[31, 32]
Neuropathic pain	Diabetic peripheral neuropathy, postherpetic neuralgia, posttraumatic nerve injury pain, etc.	Prevalence in the general population is ∼7–10%; can reach 20–30% in high‐risk groups such as diabetic patients	Frequently seen in patients with diabetes, postherpes zoster, or peripheral/central nerve injury; presents as burning, electric‐shock‐like pain and dysesthesia; higher proportions in males, the elderly, and those with metabolic syndrome	[33, 34, 35]
Migraine/tension‐type headache	Recurrent headache with nausea and photophobia (migraine); bilateral pressing/tightening headache (tension‐type)	Migraine prevalence in adults is ∼18.1%; tension‐type headache prevalence is ∼26.1%, with a substantial proportion progressing to chronicity	Migraine is most common in women aged 20–50 years; tension‐type headache is also prevalent in adolescents and young adults; both are influenced by stress, poor sleep, and hormonal fluctuations	[36, 37, 39]
Cancer‐related pain	Primary tumor pain, bone metastasis pain, treatment‐related pain(hand‐foot syndrome, eurotoxicity)	Overall prevalence of cancer pain is ∼44.5%, with ∼30.6% being moderate‐to‐severe; pain persists in ∼39.3% after curative treatment; prevalence is ∼55% during anticancer therapy; in advanced/metastatic/terminal stages, prevalence rises to ∼66.4%, with ∼38% being moderate‐to‐severe	Affects cancer patients across all ages, with the heaviest burden in advanced/metastatic stages; US NHIS estimates suggest ∼34.6% of cancer survivors experience chronic pain, with **16.1%** having high‐impact chronic pain (∼5.39 million individuals)	[40, 41, 42]

### 2.2 Risk Factors and Susceptible Populations

Chronic pain is rarely attributable to a monolithic etiology; instead, it emerges from the complex, long‐term interplay between biological susceptibility, socio‐behavioral patterns, and occupational or socio‐ecological environments. Systematically characterizing these multilevel determinants is essential for identifying susceptible populations, formulating population‐level prevention strategies, and implementing proactive, targeted interventions in high‐risk individuals.

The risk architecture of chronic pain delineates a distinct demographic and biological profile. Prevalence rises significantly with advancing age, as older adults are increasingly predisposed to persistent pain due to degenerative changes and the cumulative burden of comorbidities [43]; in one population‐based survey, approximately 30.8% of individuals aged ≥65 years reported chronic pain [44]. Several common pain phenotypes, including OA and CLBP, are notably more prevalent in middle‐aged and older cohorts, with pain intensity and duration frequently exacerbating with age [45]. However, it must be emphasized that chronic pain is not exclusive to the elderly; younger individuals are also affected, albeit at a lower overall proportion [46]. Regarding sex differences, the overall prevalence is consistently higher in women than in men [47], with chronic pain being more common among women in the general population [48]. Specifically, for conditions such as migraine, fibromyalgia, and OA, disparities in risk and severity are particularly pronounced in females [49, 50]. This divergence stems from multifactorial interactions, including biological differences in hormonal regulation and pain modulation, as well as psychosocial dimensions [51]. Genetic susceptibility further provides a “background terrain”: studies estimate the overall heritability of chronic pain at approximately 30% [52, 53], with familial aggregation being particularly evident in migraine, chronic back pain, and chronic widespread pain [54]. For instance, polymorphic variants in SCN9A and loci related to the μ‐opioid receptor are associated with hyperalgesia and susceptibility to chronic pain [55, 56], while genetic variants linked to inflammation and connective tissue may also elevate risk [56, 57].

Social and environmental dimensions equally shape the distribution and persistence of chronic pain. Lower socioeconomic status is consistently associated with chronic pain [18], where financial strain and limited resource accessibility can amplify pain experience and the risk of chronification through dual somatic and psychological pathways [58]. Prevalence is also higher among the unemployed or those with precarious employment, reflecting both pain‐induced withdrawal from the workforce and the psychological stress and barriers to healthcare access associated with unemployment itself [59], thereby forming a negative feedback loop of “poverty–pain–functional limitation.” Regarding occupational exposure, long‐term heavy manual labor, repetitive lifting, prolonged sitting or standing, and the operation of vibrating tools increase musculoskeletal load and cumulative injury, significantly elevating the risk of CLBP [60]. Consequently, the burden of low back pain is notably higher among occupational drivers, construction workers, and nursing personnel [61, 62, 63], as well as in jobs involving high repetition and poor posture [64, 65]. Ergonomic modifications, the use of assistive devices, and appropriate shift scheduling can mitigate these risks to a certain extent [66, 67].

Lifestyle factors offer modifiable entry points for intervention. Overweight and obesity are associated with an elevated risk of chronic joint and low back pain, involving both increased mechanical loading and a low‐grade inflammatory state that lowers pain thresholds and activates inflammatory mediators [68, 69]; consequently, weight management facilitates reductions in knee and low back pain intensity [70]. Smoking, beyond its systemic detriments, is linked to an increased risk of chronic pain, with potential mechanisms including vasoconstriction and tissue ischemia, intervertebral disc degeneration, enhanced inflammatory responses, and a “self‐medication” cycle involving stress and nicotine [71, 72]. Conversely, smoking cessation correlates with improvements in pain intensity and associated anxiety [73]. Physical inactivity and sedentary behavior are independent risk factors [74]; in contrast, regular, moderate‐intensity, progressive exercise helps prevent or alleviate chronic pain by strengthening musculoskeletal support, improving circulation, activating endogenous analgesic pathways, and exerting positive effects on mood and sleep [75, 76].

Psychosocial factors permeate the genesis and maintenance of chronic pain. Depression and anxiety are highly prevalent within the chronic pain population, reciprocally exacerbating one another and collectively worsening functional limitations [77]. Catastrophizing cognitions and fear‐avoidance beliefs regarding pain or movement can lead to reduced activity and deconditioning, thereby establishing a self‐reinforcing loop of “pain–avoidance–deconditioning–increased pain” [78, 79]. Furthermore, a lack of social support, major stressful life events, and adverse childhood experiences increase vulnerability [80]. Systematic psychological assessment and intervention are instrumental in disrupting these cycles, thereby enhancing the efficacy of comprehensive management.

Collectively, susceptibility to chronic pain spans biological, psychological, and social dimensions. Biologically, age‐related neurodegenerative changes, alongside sexual dimorphism in hormonal milieus and immune responses, modulate the generation, amplification, and inhibition of nociceptive signals; together with genetic background and chronic low‐grade inflammation, these factors constitute an individualized “predisposing substrate.” Societally, socioeconomic status, educational attainment, occupational burden, and lifestyle factors—such as sedentary behavior and obesity—exert persistent external pressure. Psychologically, depression, anxiety, catastrophizing, and sleep disturbances amplify and entrench the pain experience via pathways including central sensitization, fear‐avoidance, and activity reduction. Deconstructing these determinants into hierarchical levels facilitates the alignment of specific, actionable interventions—ranging from workplace ergonomic modifications, weight management, smoking cessation, and regular physical activity, to systematic psychological assessment and support, and improved accessibility and continuity of care—thereby offering a pathway to tangibly reduce the incidence and overall burden of chronic pain at the population level.

### 2.3 Socioeconomic Burden

Chronic pain is not merely a critical medical issue but also imposes a heavy and persistent socioeconomic burden, with impacts pervading direct healthcare expenditures, loss of workforce and productivity, and a systemic decline in patients' QoL.

Regarding direct costs, patients with chronic pain often require long‐term, recurrent, and multidisciplinary healthcare services—encompassing outpatient follow‐ups, diagnostic imaging, physiotherapy, surgical interventions, and ongoing pharmacotherapy—thereby driving up expenditures for both individuals and insurance systems. In Europe, for instance, the per capita annual healthcare cost for patients with chronic pain rose from approximately €3828 in 2010 to €4510 in 2016, whereas for the population without chronic pain, it increased only from €2256 to €3021 during the same period [81]. Beyond outpatient and hospitalization fees, significant components of direct expenditure include costs for analgesic medications, surgeries such as spinal fusion or joint replacement, and sustained investment in rehabilitation and assistive devices; the utilization of public health services, including community care, home nursing, and pain clinics, further intensifies the pressure on health resources [82].

The greater economic impact often stems from less visible indirect costs. Persistent pain diminishes work capacity and efficiency, leading to absenteeism and presenteeism, and compelling some patients to reduce working hours or exit the labor market prematurely. In multiple assessments, the economic losses attributed to pain‐related absenteeism, reduced productivity, and early retirement exceed those caused by most single disease entities [83]. For employers, this translates to declined performance and rising labor costs; at the societal level, it implies potential losses in Gross Domestic Product and escalating welfare expenditures, with conditions such as CLBP and arthritis being major drivers for disability claims and social assistance, incurring substantial fiscal outlays [81, 84]. Overall, while indirect costs are difficult to quantify precisely, they are widely acknowledged to be immense and often surpass direct medical costs [85].

Beyond financial implications, chronic pain exerts a profound impact on QoL. Patients frequently experience functional limitations and reduced social participation, ranging from difficulties in daily activities—such as walking, climbing stairs, lifting heavy objects, and even basic self‐care [86]—to a cascade of consequences including low mood, social withdrawal, and loss of role functioning. Primary care research indicates that over half of chronic pain patients report moderate or greater impairment: approximately 33.8% experience limitations in general physical activity, 42.3% report negative effects on mood, nearly 30% have reduced walking ability, nearly half suffer from impaired social and family relationships, one‐third endure severe sleep disturbances, and over 40% report a significantly diminished enjoyment of life [87]. Long‐term sleep disruption and accumulated fatigue further exacerbate physical and mental states [88]; in the elderly, chronic pain is also associated with cognitive decline and an increased risk of disability [89, 90].

From a public health perspective, the pressure exerted by chronic pain on healthcare systems, economic development, and social well‐being is all‐encompassing: it consumes substantial health resources, erodes labor productivity, and casts a lasting shadow over individuals and families. Elevating chronic pain to a public health priority, systematically investigating its epidemiological patterns, and deploying targeted interventions—while simultaneously validating patient needs and reducing employment discrimination and social stigma—are essential for synergistically mitigating its overall burden across medical, economic, and social dimensions.

## 3 Pathophysiology of Chronic Pain

**FIGURE 1:**
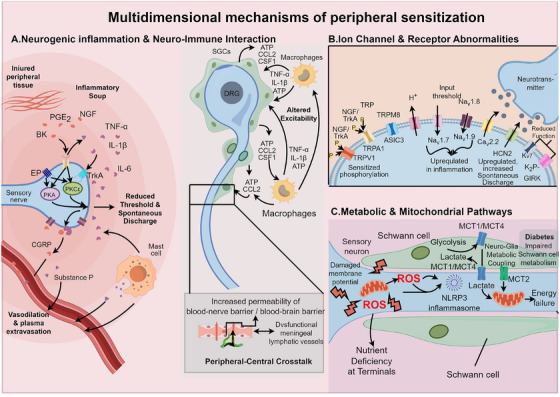
Multidimensional mechanisms of peripheral sensitization. (A) Neurogenic inflammation and neuro–immune interactions. Tissue injury in the periphery leads to the release of an “inflammatory soup,” including PGE_2_, NGF, and proinflammatory cytokines, which act on their cognate receptors on nociceptors, lowering activation thresholds and sensitizing peripheral terminals. Activated nociceptors in turn release CGRP and substance P (SP) antidromically, causing vasodilation, plasma extravasation, and mast cell activation. (B) Ion channel and receptor dysregulation. Inflammatory mediators modulate multiple ion channels via posttranslational mechanisms such as phosphorylation. The sensitivity of TRPV1, TRPA1, and ASIC3 is increased; voltage‐gated sodium channels and calcium channels are upregulated or gain function; whereas potassium channels are downregulated or functionally impaired. Together, these changes enhance membrane excitability and promote spontaneous ectopic discharges. (C) Metabolic and mitochondrial pathways. Mitochondrial dysfunction within sensory neurons results in ROS accumulation, activation of the NLRP3 inflammasome and impaired axonal transport. Schwann cells supply lactate as an energy substrate to axons via monocarboxylate transporters (MCTs); this neuron–glia metabolic coupling is disrupted in conditions such as diabetes, leading to axonal energy failure. Systemic metabolic interventions may help restore these pathways and alleviate peripheral sensitization.

**FIGURE 2:**
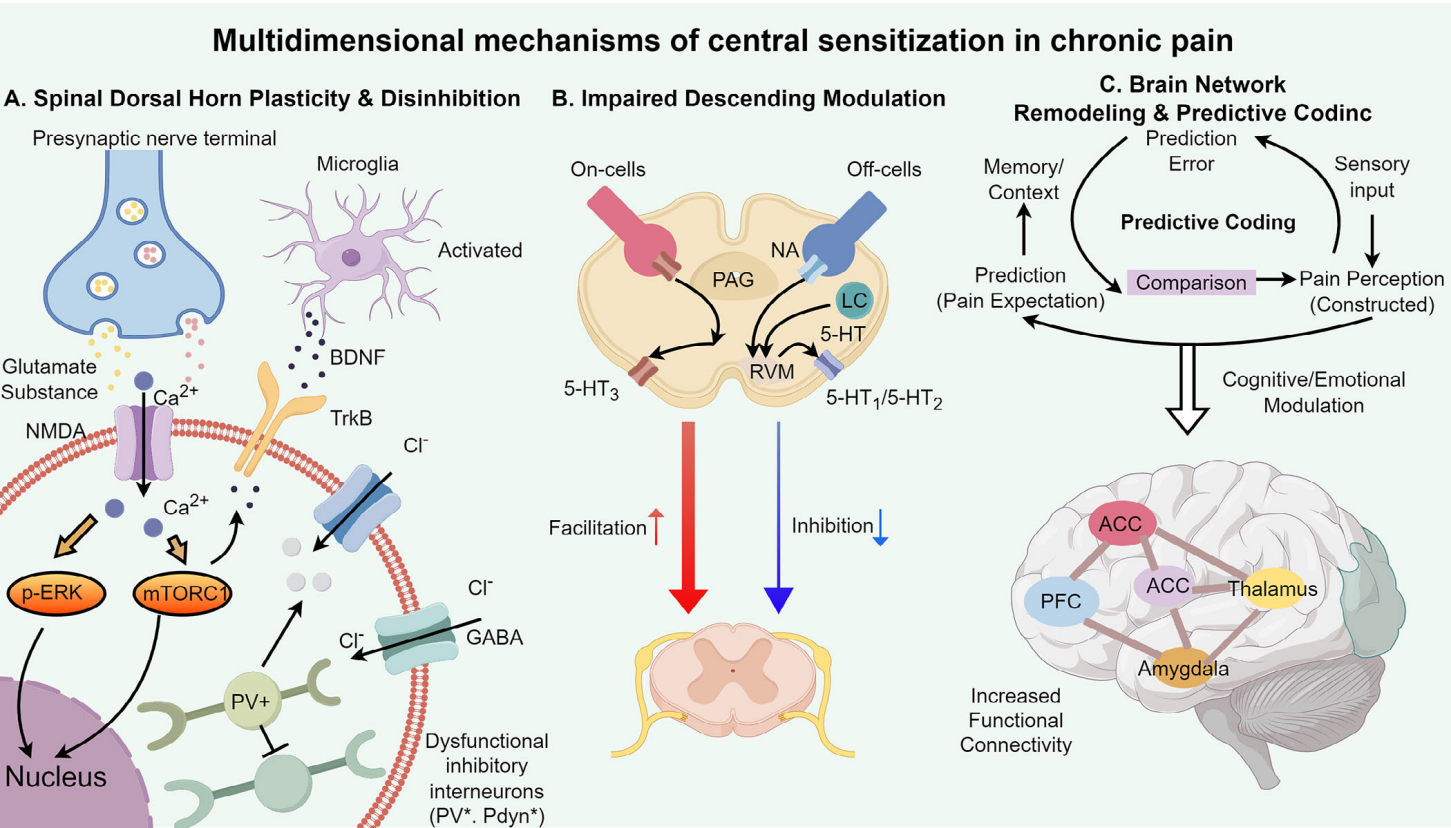
Multidimensional mechanisms of central sensitization in chronic pain. (A) Synaptic plasticity and disinhibition in the spinal dorsal horn. Persistent high‐frequency noxious input from the periphery via C‐fibers and Aδ‐fibers drives the release of glutamate and substance P at dorsal horn synapses. This activates postsynaptic NMDA receptors, relieves the Mg^2^^+^ block, and leads to pronounced Ca^2^^+^ influx. The ensuing intracellular signaling cascade, including activation of p‐ERK and mTORC1 pathways, promotes activity‐dependent protein synthesis and increases neuronal excitability. In parallel, activated microglia release brain‐derived neurotrophic factor (BDNF), which acts on neuronal TrkB receptors to trigger internalization and degradation of the K^+^–Cl^−^ cotransporter KCC2. The resulting rise in intracellular Cl^−^ converts GABA_A_ receptor‐mediated currents from hyperpolarizing to depolarizing, shifting GABAergic signaling from inhibition toward excitation. Functional loss or suppression of inhibitory interneuron populations (PV^+^, SOM^+^, Pdyn^+^) further exacerbates disinhibition and contributes to central sensitization. (B) Imbalance of descending modulatory pathways. The brainstem periaqueductal gray (PAG)–rostral ventromedial medulla (RVM) pathway, which receives inputs from higher cortical and limbic regions, exerts bidirectional control over spinal nociceptive transmission. In chronic pain, this system becomes biased toward facilitation: excitatory “On‐cells” in the RVM are hyperactive, whereas inhibitory “Off‐cells” are suppressed. Consequently, descending facilitation mediated by serotonergic and noradrenergic (NA) projections to the dorsal horn is markedly enhanced (thick red arrows), while descending inhibition is weakened (thin blue arrows), further amplifying spinal nociceptive signaling. (C) Brain network reorganization and predictive coding. Chronic pain is associated with widespread remodeling of brain networks, including strengthened functional connectivity among the anterior cingulate cortex (ACC), insula, thalamus, amygdala, and prefrontal cortex (PFC), as well as abnormal coupling with the default mode network (DMN). The inset schematizes a predictive coding framework: in chronic pain, exaggerated top–down pain predictions (priors) dominate the comparison with incoming sensory signals, so that the resulting percept can remain highly painful even when peripheral input is weak. Emotion‐ and cognition‐related circuits, particularly within the amygdala and PFC, feed into brainstem modulatory centers and bias descending control toward facilitation, establishing a self‐reinforcing loop that maintains central sensitization and persistent pain.

**FIGURE 3:**
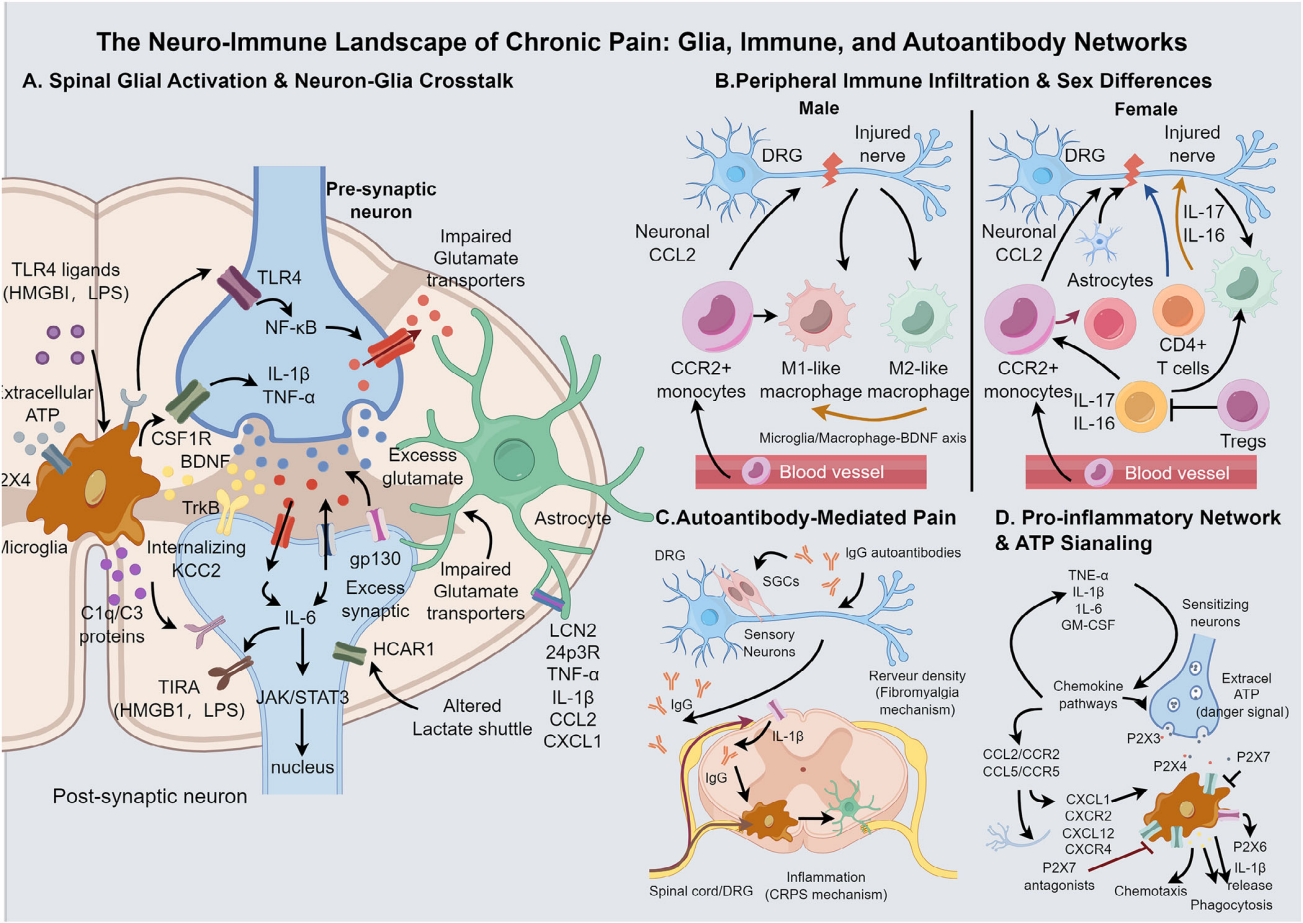
Neuro–immune landscape of chronic pain: integrated networks of glia, immune cells, and autoantibodies. (A) Neuron–glia crosstalk and activation in the spinal dorsal horn. At the synaptic level, ATP released from injured primary afferents activates P2×4 receptors on microglia, driving the release of BDNF. BDNF binds TrkB receptors on postsynaptic neurons and promotes internalization of the K^+^–Cl^−^ cotransporter KCC2, resulting in disinhibition. Neuron‐derived CSF1 stimulates microglial proliferation and a proinflammatory phenotype. TLR4 ligands activate NF‐κB signaling in microglia, leading to the release of proinflammatory cytokines (IL‐1β, TNF‐α), while complement proteins (C1q/C3) mediate preferential pruning of inhibitory synapses. In parallel, IL‐6 activates the JAK/STAT3 pathway in astrocytes; reactive astrocytes exhibit impaired glutamate uptake, altered lactate metabolism and upregulation of lipocalin‐2 (LCN2), further amplifying neuroinflammation. (B) Peripheral immune‐cell infiltration and sex‐dimorphic mechanisms. In the dorsal root ganglion (DRG) and injured peripheral nerve, neuron‐derived CCL2 recruits CCR2^+^ monocytes, which differentiate into M1‐like (proinflammatory) or M2‐like (anti‐inflammatory/reparative) macrophages. Pain mechanisms show pronounced sexual dimorphism: in males, microglia/macrophage–BDNF signaling predominates, whereas in females, T cell‐mediated mechanisms are more prominent, involving CD4^+^ T cell‐derived IL‐17 and IL‐16 and loss of regulatory T cell (Treg)‐mediated restraint. (C) Autoantibody‐mediated pain. In fibromyalgia (FM), IgG autoantibodies bind to satellite glial cells (SGCs) and sensory neurons in the DRG, leading to SGC activation and reduced intraepidermal nerve fiber density. In complex regional pain syndrome (CRPS), IgG deposition in the DRG and spinal cord engages IL‐1β‐dependent pathways, activating microglia and astrocytes and driving neuroinflammation and pain. (D) Proinflammatory mediator networks and ATP signaling as an integrative hub. Proinflammatory cytokines form self‐reinforcing loops that directly sensitize neurons and promote activation of glial and immune cells. Chemokines coordinate immune‐cell recruitment and phenotypic polarization. Extracellular ATP acts as a key danger signal, depolarizing peripheral nociceptors via P2×3 receptors and acting on spinal microglia through P2×4, P2×7, P2Y12, and P2Y6 receptors. P2×7 receptor antagonists are highlighted as promising therapeutic targets within this neuro–immune signaling network.

The genesis and maintenance of chronic pain do not stem from a single pathway but rather from a networked process involving multilevel coupling across multiple nodes. Its core manifestations are “maladaptive plasticity” of the sensory nervous system and persistent positive feedback loops driven by multisystemic dysregulation, including inflammation, immunity, and metabolism [91]. In recent years, multiscale research ranging from peripheral to central sites, and from molecular/cellular to circuit/system levels, has successively elucidated key pathways and nodes: ion channel profiles and excitability remodeling, disinhibition and the lifting of synaptic suppression, sustained glial activation, immune factor‐mediated crosstalk, metabolic reprogramming, as well as circadian rhythms and sex differences (Figures 1, 2, 3). These findings are supported by a chain of evidence comprising preclinical models, clinical studies, and multimodal biomarkers such as imaging and electrophysiology [92, 93, 94, 95, 96, 97, 98]. Crucially, these mechanistic insights are accelerating their alignment with clinical management: drugs, interventional devices, and digital therapeutics targeting specific pathological pathways are emerging continuously, gradually forming a “mechanism‐target→intervention” mapping that lays the foundation for mechanistic stratification and personalized analgesia [99]. Accordingly, this section will systematically expound upon the pathophysiology and latest advances in chronic pain, following the logic of “peripheral sensitization → central sensitization → neuro–immune interactions → genetics and epigenetics → psychosocial factors → translation and biomarkers.”

### 3.1 Peripheral Sensitization

#### 3.1.1 Nociceptor Hyperexcitability and Neurogenic Inflammation

Sustained peripheral inflammatory signals can maintain nociceptors in a state of long‐term hypersensitivity. A plethora of algesic mediators released within the milieu of tissue injury or chronic inflammation—such as prostaglandin E_2_ (PGE_2_), bradykinin, nerve growth factor (NGF), and proinflammatory cytokines like TNF‐α, IL‐1β, and IL‐6—significantly augment the excitability of peripheral primary sensory neurons, particularly nociceptors [100]. These mediators, acting via their respective receptors and downstream signaling pathways, lower the activation threshold of nociceptors and induce spontaneous discharge, thereby initiating and maintaining inflammatory hyperalgesia. For instance, PGE_2_ activation of EP receptors elevates cAMP levels, triggering protein kinase A (PKA)‐mediated phosphorylation of ion channels and reducing the pain threshold; similarly, NGF binding to tropomyosin receptor kinase A (TrkA) phosphorylates TRPV1 via pathways such as protein kinase C epsilon (PKCε), enhancing sensitivity to thermal stimuli [101]. This cascade of inflammatory factors amplifies sensitization, rendering nociceptors persistently “vulnerable,” such that stimuli normally below the pain threshold can evoke pain [102].

Furthermore, hyperexcitable nociceptors are not merely passive signal recipients; they actively release neuropeptide mediators, triggering neurogenic inflammation [103]. Classically, C‐fiber primary sensory neurons release calcitonin gene‐related peptide (CGRP) and substance P, which act on vasculature and mast cells to induce vasodilation, plasma extravasation, and tissue edema, further recruiting inflammatory cells [104]. This neuron‐mediated local inflammatory response can form a positive feedback loop with immune mediators, extending the scope and duration of inflammation. Additionally, sensory neurons engage in intimate, “entangled” interactions with surrounding non‐neuronal cells (glial and immune cells) [105]. Within peripheral sensory ganglia, such as the dorsal root ganglia (DRG), satellite glial cells (SGCs) tightly ensheath the neuronal somata; injury or inflammation activates SGCs to release signaling molecules like adenosine triphosphate (ATP) and cytokines, which act on adjacent neurons to alter their excitability [106]. Concurrently, macrophages and mast cells within the DRG are also involved: macrophages secrete proinflammatory cytokines and growth factors, while mast cells release histamine and serotonin (5‐HT), all of which collectively lower sensory neuron thresholds [107]. Conversely, injured neurons can release chemokines and colony‐stimulating factors (CSFs) to recruit and activate macrophages and SGCs [108]. This bidirectional neuro–immune communication transforms the DRG into a local inflammatory amplifier: immune cells activate neurons, triggering aberrant discharge, while active neurons further stimulate glial and immune cells, sustaining nociceptive signaling [109].

Notably, recent research has highlighted that alterations in the neurovascular unit and peripheral barrier permeability also influence crosstalk between peripheral inflammation and central sensitization. For example, chronic inflammation can increase the permeability of the blood–nerve and blood–brain barriers, facilitating the entry of peripheral inflammatory mediators and immune cells into the central nervous system [110]. Dysfunction of meningeal lymphatic vessels, which serve as a clearance route for central inflammatory products, has been shown to exacerbate pain‐related neuroinflammation, a phenomenon particularly evident in migraine models [110]. These findings suggest that peripheral and central immune‐inflammatory states interact via vascular and lymphatic networks, providing structural support for the persistence of chronic pain.

#### 3.1.2 Aberrant Ion Channels and Receptors

Transient receptor potential (TRP) channels play a pivotal role in peripheral nociception, with TRPV1, TRPA1, and TRPM8 emerging as three subtypes of particular interest. TRPV1, an ion channel sensitive to capsaicin and noxious heat, functions physiologically as a “thermal nociceptor”; however, in chronic pain states, its expression is upregulated and its sensitivity heightened. Inflammatory mediators can phosphorylate TRPV1 via PKA/PKC pathways, lowering its activation threshold such that mild thermal stimuli evoke intense pain [111, 112]. TRPA1 is responsive to a diverse array of chemical stimuli, including pungent isothiocyanates and endogenous oxidative stress products, and also participates in cold nociception; chronic inflammation can increase TRPA1 membrane expression and alter its conformation, leading to hyperalgesia [113]. TRPM8, a cold‐sensitive channel, exhibits a more complex role in neuropathic pain: some studies suggest that TRPM8 activation may alleviate pain, positioning it as a potential analgesic target [114]. Collectively, the overactivation and dysregulation of TRP family channels are considered key drivers of lowered pain thresholds in chronic conditions. Progress is being made in developing antagonists and agonists targeting these channels—for instance, TRPV1 and TRPA1 antagonists have demonstrated analgesic potential [115]. Nevertheless, given the role of TRP channels in physiological thermal sensation, achieving biased modulation—blocking pathological hypersensitivity while preserving physiological function—remains a major challenge in drug design [116].

ASIC3 acts as the predominant acid‐sensing channel in sensory neurons, exhibiting high sensitivity to pH reduction and playing a role in ischemia‐ and exercise‐induced muscle pain [117]. In patients with chronic pain, the local tissue microenvironment is frequently acidic, leading to the sustained activation of ASIC3 and persistent nociceptive signaling [118]. Animal studies have demonstrated that specific downregulation of ASIC3 via gene knockout or RNA interference significantly attenuates pain behaviors and associated hypersensitivity in models of ischemic muscle pain, suggesting that ASIC3 is a potential therapeutic target for such conditions [119]. Currently, inhibitors targeting ASICs are under investigation; for instance, amiloride and its derivatives have shown analgesic efficacy in preclinical models [117].

Peripheral primary sensory neurons express multiple subtypes of voltage‐gated sodium channels, among which Nav1.7, Nav1.8, and Nav1.9 (encoded by SCN9A, SCN10A, and SCN11A, respectively) are intimately linked to pain modulation [130]. Nav1.7 is regarded as the “threshold‐setting” channel for nociceptive signal initiation. Genetically, its variants are directly correlated with human pain phenotypes: loss‐of‐function mutations in SCN9A result in congenital insensitivity to pain, whereas gain‐of‐function mutations lead to familial episodic pain syndromes—such as inherited erythromelalgia and paroxysmal extreme pain disorder—or small fiber neuropathy, causing unbearable spontaneous pain [120]. Pathophysiologically, chronic inflammation and nerve injury can upregulate Nav1.7 expression in the DRG and peripheral axons and alter its electrophysiological properties, rendering neurons more prone to discharge [121]. Consequently, Nav1.7 has become a premier target for analgesic drug development, with multiple selective blockers entering clinical trials, including small‐molecule inhibitors and engineered biotoxins that anchor to the channel [122]. Nav1.8 and Nav1.9 primarily mediate sustained currents in sensory neurons and play prominent roles in inflammatory pain; for instance, inflammatory mediators can upregulate Nav1.8 and Nav1.9 expression via the p38 MAPK pathway. Selective blockers of Nav1.8 have demonstrated analgesic efficacy in models of diabetic neuropathy, while Nav1.9, owing to its unique current properties, is being investigated as a potential target with a favorable side‐effect profile [123, 124].

Regarding calcium channels, N‐type voltage‐gated calcium channels (CaV2.2), located at sensory nerve terminals and within the spinal dorsal horn, mediate neurotransmitter release; their overactivation amplifies nociceptive signal transmission [125]. Clinically, drugs targeting CaV2.2, such as the conotoxin derivative ziconotide, are utilized for intrathecal analgesia in intractable pain [126], while novel oral CaV2.2 blockers are currently under development. In the context of HCN channels, hyperpolarization‐activated cyclic nucleotide‐gated cation channels contribute to maintaining resting excitability in sensory neurons. Following nerve injury, HCN channel expression is upregulated, predisposing neurons to spontaneous discharge; notably, HCN2‐deficient mice exhibit attenuated hyperalgesia [127]. As for potassium channels, various subtypes—including voltage‐gated Kv7, two‐pore domain K_2_P, and G protein‐coupled inwardly rectifying GIRK channels—typically exert homeostatic hyperpolarizing and inhibitory effects on neurons. In chronic pain states, the function or expression of certain K^+^ channels is diminished, leading to neuronal hyperexcitability [128]. Enhancing potassium channel function can induce analgesia; for instance, Kv7 openers have been employed to treat neuralgia, although their use has been limited by side effects [129, 130, 131]. GIRK channels serve as downstream effectors for numerous analgesic receptors, and their dysfunction is implicated in opioid tolerance and hyperalgesia [132]. In summary, the aberration of ion channels and receptors is pivotal in peripheral sensitization and provides a multitude of targets for analgesic intervention.

#### 3.1.3 Metabolic and Mitochondrial Pathways

The mitochondrial status of peripheral sensory neurons exerts a significant influence on their excitability. In chronic pain models, impaired mitochondrial function, reduced membrane potential, and excessive production of reactive oxygen species (ROS) are frequently observed in sensory neurons [133]. Excessive ROS can directly modify ion channels and receptors, heightening nociceptor sensitivity, and can further amplify pain through the activation of inflammatory signaling [134]. Furthermore, mitochondrial dysfunction is often accompanied by aberrant axonal transport and insufficient nutrient supply to sensory nerve terminals, thereby leading to nerve fiber degeneration and the abnormal amplification of pain signals [135]. Interventions targeting mitochondria have demonstrated potential in animal models: antioxidants and agents that improve mitochondrial electron transport have been shown to attenuate neuropathic pain behaviors to a certain degree [136].

Beyond neuronal metabolism itself, neuro–glial metabolic coupling serves as a critical foundation for maintaining peripheral nerve function. Schwann cells rely primarily on glycolysis to generate lactate, which is exported via monocarboxylate transporters MCT1 and MCT4; subsequently, lactate is taken up by MCT2 at the axonal terminal and enters mitochondria for oxidative phosphorylation, thereby providing energy for long‐distance axonal conduction and terminal activity [137]. In mice with Schwann cell‐specific deletion of Mct1, while myelin structure remains largely intact, neuromuscular junctions at motor endplates progressively degenerate, indicating that the metabolic support of lactate supply from Schwann cells to axons is vital for the long‐term maintenance of peripheral nerve terminals [138]. Diabetic peripheral neuropathy is viewed as a prototypical state of “bioenergetic imbalance”: insulin resistance, hyperglycemia, and lipotoxicity can impair mTORC1 signaling in Schwann cells and potentially compromise MCT‐mediated lactate export. Simultaneously, this leads to axonal mitochondrial depolarization and ROS accumulation, causing energy failure and degeneration initially in distal axons, a process closely linked to the development of painful diabetic neuropathy. These findings provide a theoretical basis for designing mechanism‐based analgesic strategies focused on “maintaining or restoring neuro–glial metabolic coupling.”

At the level of systemic metabolic intervention, a randomized controlled clinical trial demonstrated that a ketogenic diet, characterized by strict carbohydrate restriction, resulted in greater pain reduction, weight loss, and improvements in depression and anxiety compared with a general whole‐food diet in patients with chronic musculoskeletal pain, accompanied by decreased levels of the inflammatory marker hsCRP [139]. This suggests that systematically improving energy metabolism and inflammatory status holds promise as an adjunctive strategy for controlling chronic pain. Overall, existing evidence underscores that the “mitochondria–ROS–inflammation/NLR family pyrin domain containing 3 (NLRP3) pathway” and “Schwann cell–axon metabolic coupling” collectively participate in the genesis and maintenance of chronic pain, providing crucial leads for mechanism‐based analgesic strategies targeting metabolic remodeling [134].

### 3.2 Central Sensitization

#### 3.2.1 Spinal Dorsal Horn

Under conditions of chronic pain, synaptic plasticity changes analogous to long‐term potentiation occur within the spinal dorsal horn. Sustained high‐frequency nociceptive input activates NMDA receptors, particularly the NR2B subtype, and removes the magnesium ion block, triggering a cascade of calcium influx [140]. This leads to the persistent activation of intracellular signaling pathways such as mTOR and ERK, promoting excitatory neurotransmitter release and postsynaptic sensitization, thereby amplifying incoming pain signals [141]. Studies indicate that levels of phosphorylated ERK (p‐ERK) in spinal dorsal horn neurons are significantly elevated in pain states, and blockade of the ERK pathway can attenuate hyperalgesia [142]. Similarly, aberrant activation of mTORC1 facilitates local protein synthesis and synaptic structural remodeling, effectively allowing pain transmission pathways to “memorize” the trace of noxious stimuli [141]. These molecular events collectively result in a lowered threshold for spinal excitability and increased response gain, causing normal peripheral signals to be excessively amplified at the spinal level.

Under normal physiological conditions, inhibitory interneurons in the spinal dorsal horn maintain robust lateral inhibition via GABA_A and glycine receptors, preventing the excessive spread of nociceptive signaling [143]. However, nerve injury or chronic inflammation can compromise this inhibitory control, with one mechanism involving the disruption of chloride homeostasis mediated by the brain‐derived neurotrophic factor (BDNF)–TrkB–KCC2 pathway [144]. Following injury, activated microglia release BDNF, which acts on TrkB receptors on spinal dorsal horn neurons to downregulate the expression and function of the potassium‐chloride cotransporter KCC2 [145]. KCC2 is responsible for extruding intracellular Cl^−^ to maintain a low‐chloride environment; its impairment leads to an accumulation of intracellular Cl^−^[143]. Consequently, upon the opening of Cl^−^ channels by GABA or glycine receptors, Cl^−^ may no longer flow inward (hyperpolarization) but instead flow outward or exhibit reduced influx (depolarization), thereby significantly attenuating the inhibitory effect of GABA/glycine or even converting it to excitation [145]. This disinhibition is considered a key hallmark of central sensitization [146]. In animal models, knockdown of spinal KCC2 induces hyperalgesia, whereas restoring KCC2 function alleviates neuropathic pain [147]. Notably, recent research has further elucidated the molecular details by which BDNF promotes the ubiquitination and degradation of KCC2 to reduce its membrane expression, providing leads for developing analgesic strategies aimed at preserving KCC2 function [148].

The spinal dorsal horn harbors various types of interneurons, a subset of which are inhibitory, including molecularly defined subtypes such as Parvalbumin (PV)‐positive, Prodynorphin (Pdyn)‐positive, Somatostatin (SOM)‐positive, and Neuropeptide Y‐positive neurons [149]. In states of chronic pain, distinct inhibitory subtypes exhibit differential vulnerability. For instance, PV‐positive interneurons typically modulate non‐nociceptive tactile input, preventing it from activating pain pathways; following nerve injury, the firing frequency of these neurons is significantly reduced, and the synchronization they mediate within the network is disrupted [150]. Intracellular recording and multielectrode array studies have demonstrated that after nerve injury, the firing pattern in the spinal dorsal horn shifts from synchronous fine coding to a desynchronized, disordered state, a transition driven precisely by the loss of PV inhibitory neuron function. Selectively silencing PV‐positive interneurons can mimic the mechanical allodynia induced by nerve injury, whereas activating or restoring their function can partially reverse hypersensitive behaviors [117]. This suggests that the “absence” of inhibitory cells such as PV neurons is critical for mechanical allodynia [150]. Similarly, Pdyn‐positive inhibitory neurons are posited to play a role in the “gating” of tactile and nociceptive signals. An optogenetic study targeting Pdyn‐positive neurons revealed that in chronic pain, these neurons become abnormally sensitive to Aβ‐fiber input, potentially recruiting innocuous tactile signals into pain pathways due to synaptic reorganization [149]. Collectively, chronic pain is accompanied by the functional loss or reorganization of specific inhibitory interneuron subpopulations, constituting a pivotal mechanism of central sensitization, particularly disinhibition. This finding also implies that protecting or restoring these inhibitory circuits holds promise as an intervention for chronic hyperalgesia.

#### 3.2.2 Descending Modulation Pathways

The periaqueductal gray (PAG)–rostral ventromedial medulla (RVM) pathway is central to the descending modulation of pain. The PAG receives input from higher brain centers and exerts bidirectional control over spinal nociceptive transmission via the RVM: encompassing both descending inhibition and descending facilitation. Under physiological conditions, inhibition and facilitation maintain a dynamic equilibrium to adapt pain responses to varying contexts; however, in chronic pain, this balance often shifts toward enhanced descending facilitation and diminished descending inhibition. The RVM contains two populations of pain‐modulating neurons: “On‐cells” and “Off‐cells.” The former discharge during noxious stimulation and facilitate pain transmission, whereas the discharge of the latter inhibits nociception [151]. In chronic pain models, enhanced activity of RVM On‐cells and suppression of Off‐cells are observed, correlating with persistent behavioral hypersensitivity [152]. Neurochemically, serotonergic and noradrenergic pathways projecting from the RVM to the spinal cord play major roles. 5‐HT exerts opposing effects via distinct receptors: activation of 5‐HT_3_ receptors facilitates spinal pain transmission, whereas activation of receptors such as 5‐HT_1_/5‐HT_7_ inhibits nociceptive signals [153]. Noradrenaline primarily acts on presynaptic α_2_‐adrenergic receptors in the spinal cord to inhibit primary afferent release, producing potent analgesia [154]. In chronic pain states, dysfunction of structures such as the nucleus raphe magnus within the RVM alters 5‐HT release patterns, leading to a predominance of facilitatory 5‐HT signaling and attenuated inhibitory signaling, resulting in insufficient descending inhibition and excessive facilitation [155]. Concurrently, descending noradrenergic inhibition originating from the locus coeruleus is also frequently weakened, further exacerbating hyperalgesia [154]. This elucidates why drugs such as 5‐HT–noradrenaline reuptake inhibitors (SNRIs) can alleviate chronic pain: they enhance synaptic concentrations of 5‐HT and noradrenaline, thereby partially restoring descending inhibitory function [156].

Descending modulation is not autonomously executed by the brainstem alone but is significantly influenced by higher brain centers governing emotion and cognition. Chronic pain is frequently accompanied by emotional states such as anxiety and depression, one neural mechanism of which is the aberrant regulation of descending pathways by the limbic system [157, 158]. Limbic–cognitive regions, including the anterior cingulate cortex (ACC), insula, amygdala, and prefrontal cortex, participate in the affective and attentional evaluation of pain and influence descending pain modulation via structures such as the PAG [159]. In chronic pain, particularly in patients with severe emotional disturbances and functional impairment, connectivity between the ACC–insula and the midbrain PAG often undergoes reorganization: functional imaging reveals enhanced resting‐state activity, suggesting the formation of an “interlocked circuit” between pain and negative affect [160]. The consequence of this circuitry is sustained attention to pain and intensified fear and expectation of pain, which further amplifies nociceptive signals via descending pathways [161]. Accordingly, psychological interventions such as cognitive behavioral therapy (CBT) and neuromodulation techniques, by regulating these higher‐order circuits, can alleviate chronic pain intensity and emotional distress to a certain degree [162, 163]. In summary, central descending modulation pathways undergo a “defense‐to‐offence” shift in chronic pain—attenuated inhibitory control and enhanced facilitatory influence—while emotional and cognitive factors drive this imbalance, creating a vicious cycle where pain fuels tension and tension exacerbates pain [164].

#### 3.2.3 Brain Network Remodeling and Predictive Coding

Chronic pain is not confined to spinal mechanisms; prolonged pain experience induces plastic reorganization across multiple functional brain networks. Studies demonstrate that connectivity patterns among the default mode network (DMN), central executive network, and affective network differ significantly between patients with chronic pain and healthy individuals [165]. For instance, in patients with chronic back pain and fibromyalgia, resting‐state functional magnetic resonance imaging (fMRI) reveals aberrant coupling between the DMN and pain networks, suggesting that the brain recurrently processes pain‐related information even at rest [166]. Concurrently, enhanced functional connectivity within the thalamo–limbic system, particularly between the thalamus and the insula/ACC, is considered the central basis for sustained nociceptive arousal and emotional distress [167]. Structural imaging also identifies alterations in gray matter density in patients with chronic pain, such as atrophy in the ACC and hippocampus and hypertrophy in the sensory cortex, likely reflecting “use‐dependent” plasticity under long‐term nociceptive stimulation [168]. These alterations result in an increased cognitive–affective weighting of pain, rendering patients more sensitive to pain and less able to distract their attention from it [169]. Notably, these cerebral changes exhibit plasticity and partial reversibility: some studies report that following successful analgesic treatment, brain functional connectivity and morphology can trend toward normalization [170]. Thus, alterations in brain networks are both a consequence of chronic pain and a critical maintenance factor for its persistence.

Modern pain neuroscience has introduced predictive coding theory to elucidate certain phenomena in chronic pain. This theory posits that the brain does not passively receive sensory information but actively predicts inputs and adjusts perception based on prediction errors [116]. In the context of pain, the brain generates predictions regarding intensity and location based on memory and context, subsequently comparing these with peripheral inputs to form the final pain experience [171]. In chronic pain, owing to the consolidation of long‐term pain memories, the brain may develop an erroneous internal model; that is, it continues to “expect” pain even when peripheral noxious stimuli have diminished or ceased, thereby maintaining the perception of pain [172]. In such scenarios, pain perception becomes partially decoupled from peripheral drive, transforming into an endogenously constructed phenomenon [173]. For example, phantom limb pain in some amputees is thought to relate to the brain's pain predictions for the missing limb; similarly, patients with CLBP who experience pain without obvious tissue injury may do so because of persistent cerebral predictive signals constructing the pain experience [174]. The predictive coding framework also explains the modulation of pain by expectation and attention: positive expectations of analgesia can be viewed as the brain lowering the predicted intensity of pain, resulting in reduced perceived pain; conversely, fear and negative expectations elevate the predicted value of pain, leading to intensified pain experience [175]. Neuroimaging studies reveal that during placebo analgesia, the prefrontal–midbrain pathway is active with increased endogenous opioid release, whereas nocebo effects activate anxiety‐related regions such as the amygdala and release cholecystokinin (CCK) to antagonize endorphins—neural manifestations of how prediction shapes pain perception [176].

To quantify the degree of central sensitization, researchers have endeavored to identify imaging and electrophysiological biomarkers. Currently promising indicators include resting‐state functional connectivity fingerprints [177]; the cortical excitation/inhibition ratio [178]; translocator protein (TSPO)–PET imaging [179]; and magnetic resonance spectroscopy (MRS) for detecting neurochemical levels [180]. However, the reproducibility and specificity of these potential biomarkers remain under validation [181]. Inconsistencies persist across studies, and diagnostic accuracy at the individual level remains insufficient. Future large‐sample, multicenter studies are required to confirm their reliability and facilitate their translation into clinical pain phenotyping and therapeutic monitoring [182].

### 3.3 Neuro–Immune Interactions

#### 3.3.1 Glial Cells in the Spinal Cord and Brain

In chronic pain states, particularly neuropathic pain, microglia within the spinal dorsal horn transition from a resting to an activated state, releasing a variety of sensitizing mediators and modulating neuronal function. A classic pathway of microglial activation involves the P2×4 receptor: following nerve injury, microglia upregulate the ATP receptor P2×4. ATP released from surrounding injured neurons and glia activates P2×4, triggering microglia to release BDNF and other factors, thereby initiating the aforementioned BDNF–TrkB–KCC2‐mediated central disinhibition [183]. Mice with P2×4 receptor blockade or gene deletion exhibit significantly attenuated hyperalgesia after nerve injury, validating the criticality of this pathway [184]. Another pivotal mechanism involves the CSF1R receptor; injured DRG neurons release CSF1 into the spinal cord, which binds to CSF1R on the microglial surface, inducing microglial proliferation and a proinflammatory phenotype [185]. Administration of CSF1R inhibitors can selectively deplete microglia, thereby suppressing postinjury hyperalgesia [186]. Furthermore, microglia sense certain damage signals via TLR4 receptors—such as the damage‐associated molecular pattern high mobility group box 1 (HMGB1), bacterial endotoxin lipopolysaccharide, and even high‐dose morphine—activating downstream NF‐κB and releasing proinflammatory cytokines like IL‐1β and TNF‐α to exacerbate pain [187]. The complement system is also implicated: microglia can secrete complement components C1q/C3 to mediate excessive synaptic pruning, particularly of inhibitory synapses, which is considered a component of central network reorganization in chronic pain [188, 189]. Collectively, microglia amplify pain signals via multiple pathways: directly releasing algesic substances such as IL‐1β and BDNF, and phagocytosing or pruning neuronal synapses to alter circuit excitation/inhibition balance.

Astrocytes also exhibit reactive changes in chronic pain. Under chronic pain conditions, astrocytes in the spinal cord and brain undergo proliferation characterized by high GFAP expression, accompanied by altered secretion of various cytokines and neurotrophic factors [190]. The JAK/STAT3 pathway is identified as a critical mechanism for astrocyte‐mediated maintenance of chronic pain: following nerve injury, proinflammatory mediators such as IL‐6 activate gp130 receptors on the astrocyte surface, triggering JAK kinase activation and STAT3 phosphorylation, which promotes the transcription of a suite of proinflammatory genes [191]. In animal models, inhibition of intracellular STAT3 signaling in astrocytes significantly reverses late‐phase neuropathic pain behaviors, indicating that the “maintenance activation” of astrocytes is crucial for the persistence of chronic pain [192]. Astrocytes also influence neuronal metabolism: their physiological functions include clearing glutamate from the synaptic cleft to prevent excitotoxicity via the glutamine cycle and fueling neurons through the astrocyte–neuron lactate shuttle [193]. In chronic pain, these functions may become dysregulated—for instance, diminished glutamate uptake capacity leads to local spinal glutamate accumulation and persistent excitation [194]; or aberrant lactate export may cause neuronal energy deficits or alter neuronal excitability via HCAR1 signaling as previously mentioned [195]. Furthermore, under conditions of neuroinflammation and nerve injury, multiple cell types including microglia, reactive astrocytes, and neurons can induce the expression and secretion of lipocalin‐2 (LCN2). LCN2 acts on microglia via its receptor 24p3R to induce the expression of TNF‐α, IL‐1β, and proalgesic chemokines such as CCL2 and CXCL1, thereby promoting microglial activation and the development of neuropathic pain [196, 197, 198]. Thus, astrocytes are not mere bystanders but, together with microglia and neurons, form a complex tripartite interaction network within chronic pain circuitry, collectively sustaining the pain state [199].

#### 3.3.2 Peripheral Immunity and Autoantibodies

A mechanism of T cell‐mediated sexual dimorphism was proposed in the mid‐2010s. Sorge et al. discovered that in various models of neuropathic pain, mechanical allodynia in male mice is dependent on spinal microglia, with inhibition or depletion of microglia reversing hypersensitivity; in contrast, females maintain a similar degree of mechanical allodynia in the absence of microglial involvement, relying instead on adaptive immune cells, particularly T lymphocytes [132]. Subsequently, Kuhn et al. further demonstrated that following peripheral nerve injury, regulatory T cells (Tregs) in the spinal cord of female mice suppress CSF1‐induced microglial activation and hyperalgesia; depletion of Tregs renders females “sensitive” to microglia‐targeted interventions once again, supporting an immunological division of labor model where “males rely predominantly on microglia and females on T cells” [200].

At the effector molecule level, CD4^+^ T cells and their cytokines provide critical proalgesic drive for neuropathic pain in females. In nerve injury models, IL‐17 has been shown to participate in spinal synaptic plasticity and the maintenance of neuropathic pain, with blockade of the IL‐17/IL‐17 receptor axis attenuating mechanical allodynia, suggesting the T cell–IL‐17 axis as a significant proalgesic pathway [201]. More recent research indicates that in female mice, CD3^+^CD4^+^ T cells activate spinal astrocytes via IL‐16–CD4 signaling to sustain neuropathic pain; intervention targeting IL‐16 or CD4 selectively alleviates hypersensitivity in females [202]. By contrast, the microglia–neuron pathway remains central in male models: microglia‐derived BDNF in the primary somatosensory cortex mediates neuronal plasticity and mechanical allodynia [203]; the CSF1R inhibitor pexidartinib, by depleting spinal microglia and DRG macrophages, produces significant analgesia in male mice, whereas its efficacy is markedly reduced in females and castrated males, demonstrating a sex‐ and androgen‐dependent difference [204]. Collectively, males rely more heavily on the microglia–BDNF axis, while females depend more on T cells and their cytokine pathways, providing a solid experimental basis for incorporating sex stratification into future analgesic drug development and clinical trial design.

During the genesis and development of chronic pain, peripheral blood monocytes can traverse the blood–nerve barrier, infiltrating injured nerves and DRG. There, they differentiate into macrophages and, alongside resident macrophages, coregulate neuroinflammation and neuronal excitability [205, 206]. Single‐cell and spatial transcriptomics studies have revealed the existence of functionally heterogeneous macrophage subpopulations within the DRGs and peripheral nerves: one subset exhibits a proinflammatory phenotype with high expression of CCR2, TNF, and IL1B, while another is enriched in molecules such as Arg1 and IL‐10, biasing toward a tissue repair/anti‐inflammatory phenotype [207]. In models of neuropathic and OA pain, proinflammatory macrophages expand significantly within the DRG; their secretion of TNF‐α and IL‐1β enhances sensory neuron discharge and maintains mechanical allodynia. Conversely, inducing macrophage polarization toward an M2/anti‐inflammatory phenotype or the exogenous administration of M2‐like macrophages alleviates persistent pain and neuroinflammation [208, 209]. The CCL2–CCR2 axis is a critical pathway driving monocyte recruitment and polarization: sensory neurons and glial cells upregulate CCL2, guiding CCR2^+^ monocytes into the DRG and promoting their differentiation toward a proinflammatory phenotype; accordingly, CCR2 antagonists or CCL2 neutralization reduce macrophage infiltration and attenuate hyperalgesia [210]. These findings suggest that finely dissecting the lineage and plasticity of distinct macrophage subpopulations, and targeting pathways such as CCR2 or promoting IL‐10‐high reparative macrophages, holds promise for achieving more precise immunomodulatory analgesic strategies [209].

The causal role of autoimmune antibodies in certain chronic pain syndromes has recently been supported by compelling evidence. Most notably in fibromyalgia, a study published in the *Journal of Clinical Investigation* demonstrated that passive transfer of IgG from fibromyalgia patients to mice induced widespread mechanical and cold hypersensitivity, reduced locomotor activity, decreased muscle strength, and reduced intraepidermal nerve fiber density. In contrast, IgG from healthy controls or IgG‐depleted serum failed to produce similar effects, suggesting the presence of pronociceptive autoantibodies within patients' IgG [101]. Histological analysis further revealed that these pathogenic IgG antibodies primarily deposited in the DRG, particularly surrounding SGCs and a subset of sensory neurons, supporting the view that “peripheral sensory ganglia are primary targets of attack” [101]. Similar passive transfer experiments have been validated in complex regional pain syndrome (CRPS): IgG from CRPS patients, in the context of mild trauma, elicited persistent pain and local inflammation in mice, inducing significant activation of spinal microglia and astrocytes. Part of this effect was dependent on IL‐1β signaling, as IL‐1 receptor antagonists could prevent or reverse the nociceptive phenotype [211, 212]. Building on this, scholars have proposed that “an autoimmune‐driven subgroup exists within the spectrum of unexplained chronic pain,” systematically summarizing evidence of autoantibodies against neural or connective tissue structures in patients with fibromyalgia, CRPS, and subsets of CLBP and chronic fatigue syndrome. This provides a theoretical rationale for trialing immunotherapies such as intravenous immunoglobulin, plasma exchange, or B‐cell depletion [213]. Collectively, these works redefine certain chronic pain conditions from traditional “functional” disorders to potential antibody‐mediated diseases, suggesting that future efforts must identify specific antigen targets and establish biomarkers to screen for “autoimmune‐type pain” patients, thereby enabling personalized immunomodulatory analgesia.

#### 3.3.3 Proinflammatory Mediator Networks

Within the neuro–immune crosstalk of chronic pain, proinflammatory cytokines and chemokines form a complex self‐reinforcing network. Key proinflammatory factors such as TNF‐α, IL‐1β, IL‐6, and GM‐CSF are elevated in affected nerves and the spinal cord of patients with chronic pain. These factors can act directly on sensory neurons and glial cells, enhancing their excitability and inducing further inflammation. For instance, TNF‐α activates neuronal Na^+^ and Ca^2^^+^ channels via the TNFR1 receptor, rendering neurons more vulnerable [214]; IL‐1β promotes glutamate release while reducing GABA release from dorsal horn neurons [215]. GM‐CSF has been identified as a critical mediator in the chronification of inflammatory pain: blockade of GM‐CSF or its receptor prevents the transition of arthritic pain to a chronic state [216]. Regarding chemokines, the CCL2/CCR2 axis participates in neuroinflammation and pain maintenance by recruiting and activating CCR2^+^ monocytes and microglia [217]. The CCL5 (RANTES)–CCR5 axis is similarly upregulated in neuropathic pain; blockade of CCR5 or dual CCR2/CCR5 antagonism attenuates mechanical allodynia, suggesting the potential therapeutic value of targeting this axis within neuro–immune pain circuitry [218]. Besides regulating peripheral neutrophils, the CXCL1/CXCR2 axis also contributes to central pain maintenance: in a model of radicular pain induced by nucleus pulposus, spinal dorsal horn astrocytes upregulate CXCL1, activating neuronal CXCR2 to sustain mechanical and thermal hypersensitivity [219]. The CXCL12/CXCR4 axis has been proven crucial for pain maintenance across various inflammatory and cancer pain models; the CXCR4 antagonist AMD3100 can reverse established hyperalgesia, involving mechanisms such as DRG SGC–neuron crosstalk and the spinal CXCR4–RhoA/ROCK2 pathway [220, 221, 222].

ATP, functioning as a “danger signal” of cellular injury and stress, widely participates in inflammation and neuromodulation within peripheral and central pain circuits [223]. Injured tissues, as well as activated glia and neurons, release ATP into the extracellular space, where it acts on two classes of purinergic receptors: ligand‐gated ion channels (P2X receptors) and G protein‐coupled receptors (P2Y receptors). At sensory nerve terminals, ATP primarily induces depolarization via P2×3 and P2×2/3 receptors to generate nociception [224]. In the spinal cord, activation of P2×4 receptors on microglia triggers BDNF release, while P2×7 receptor activation induces NLRP3 inflammasome assembly and IL‐1β secretion [225, 226]. Among P2Y receptors, P2Y12 plays a significant role in microglial chemotaxis, enabling microglia to sense ATP gradients and migrate toward lesion sites; P2Y6 mediates microglial phagocytosis of synaptic debris. Collectively, ATP–purinergic signaling permeates multiple stages of pain genesis, from peripheral inflammatory pain to central neuropathic pain. Its broad spectrum of activity makes it a compelling target for analgesic intervention [227] P2×7 antagonists have demonstrated analgesic efficacy in inflammatory and neuropathic pain models, and clinical trials are currently evaluating their feasibility for treating chronic pain [228].

### 3.4 Genetics and Epigenetics

#### 3.4.1 Genetic Susceptibility

Population genetic studies have unveiled specific genetic variants associated with pain sensitivity. Variations in SCN9A (Nav1.7) are paradigmatic: biallelic loss‐of‐function mutations result in congenital insensitivity to pain, whereas gain‐of‐function mutations lead to inherited hyperalgesia syndromes, such as familial erythromelalgia and paroxysmal extreme pain disorder, characterized by severe burning pain or paroxysmal attacks [120, 229]. These extreme phenotypes have established SCN9A as a preeminent “pain gene.” Furthermore, common single nucleotide polymorphisms in SCN9A may influence pain sensitivity in the general population; for instance, a specific SCN9A haplotype has been linked to altered pain thresholds in red‐haired individuals [55]. The Val158Met polymorphism in COMT (catechol‐O‐methyltransferase) is another well‐characterized variant associated with pain susceptibility and opioid response: the Met allele confers reduced *COMT* activity and decreased catecholamine degradation, with carriers reportedly exhibiting higher pain scores and diminished morphine analgesia [230, 231]. The A118G polymorphism in OPRM1 (μ‐opioid receptor) affects receptor affinity, thereby modulating pain and opioid effects: the G allele reduces receptor expression, resulting in higher morphine requirements and slightly weaker analgesic efficacy [232]. Rare variants in TRPA1 have also been reported; a point mutation in the TRPA1 gene was found in a pedigree where young members suffered from episodic debilitating pain, linked to aberrant TRPA1 channel activation [233]. These findings of monogenic or low‐frequency variants demonstrate a clear genetic basis for nociception, explaining some interindividual variability in pain sensitivity. However, for most common chronic pain conditions, the genetic determinants are complex and polygenic.

Recent large‐scale genome‐wide association studies have endeavored to identify susceptibility loci for chronic pain. For instance, analyses of UK Biobank data concerning multisite chronic pain have uncovered several significant loci involving genes related to neurodevelopment, synaptic plasticity, and immune regulation [234]. Although the effect size of individual loci is small, incorporating dozens of loci into a polygenic risk score (PRS) enables the differentiation of high‐risk groups at the population level [235]. A 2022 study constructed a PRS capable of predicting the risk of developing widespread chronic pain to a certain degree [236]. Nevertheless, the current predictive accuracy of PRS for individuals remains limited, as environmental factors and gene–environment interactions exert a profound influence on chronic pain. Nonetheless, these achievements demonstrate that chronic pain is a complex trait determined by multiple loci with small effects. With larger sample sizes and deeper functional investigations, genetic information holds promise for screening susceptible populations or guiding the selection of more effective therapies for distinct patient subgroups [237].

#### 3.4.2 Epigenetics and RNA Modification

While genetic factors dictate the fundamental architecture of the “pain circuit,” epigenetic mechanisms persistently regulate gene expression without altering the DNA sequence, serving as the molecular basis for chronic pain “memory” [238]. Animal model studies have revealed significant alterations in the DNA methylation status of promoter regions of pain‐related genes following nerve injury: certain genes that inhibit nociception become silenced via hypermethylation, whereas genes promoting hypersensitivity are activated through demethylation [239]. The use of DNA methyltransferase inhibitors can block or reverse neuropathic pain behaviors, demonstrating the indispensability of DNA methylation in the maintenance of chronic pain [240]. Concurrently, histone acetylation/deacetylation plays a role; for instance, the upregulation of histone deacetylases (HDACs) in spinal dorsal horn neurons can suppress the expression of analgesic genes, while administration of HDAC inhibitors attenuates chronic hypersensitivity [241]. Notably, different HDAC subtypes exert distinct effects: some studies suggest that HDAC1/2 play a significant role in maintaining hypersensitivity, whereas inhibition of HDAC3 yields less pronounced analgesic effects [242]. Furthermore, chromatin remodeling complexes and histone methylation have also been implicated; for example, upregulation of H3K27 trimethylation mediated by the enhancer EZH2 has been found to participate in gene repression during neuropathic pain states, and EZH2 inhibition alleviates pain [243]. These epigenetic modifications maintain the nervous system in a “hypersensitive pain state” even after the cessation of noxious signaling, thereby forming a so‐called “pain memory.”

Accumulating evidence indicates that noncoding RNAs play a pivotal regulatory role in chronic pain. Among them, microRNAs (miRNAs)—small RNAs approximately 22 nucleotides in length—bind to target mRNAs to repress their translation [244]. Dysregulation of various miRNAs has been demonstrated in pain models: for instance, miR‐146a and miR‐155 are upregulated following nerve injury, promoting proinflammatory cytokine expression; inhibition of these miRNAs attenuates inflammatory and neuropathic pain behaviors [245]. Conversely, some miRNAs, such as miR‐219, are downregulated in chronic pain, leading to increased expression of NMDA receptor subunits; intrathecal administration of miR‐219 mimics reduces hypersensitivity [246]. Consequently, scholars have developed antisense oligonucleotides (antagomirs) or mimics targeting miRNAs to modulate pain‐related gene expression. For example, antisense sequences targeting miR‐21 have demonstrated analgesic efficacy in neuropathic pain models [247]. Beyond miRNAs, long noncoding RNAs and circular RNAs also participate in chronic pain regulation, acting as “sponges” to adsorb miRNAs or forming complexes with proteins to alter transcription, thereby influencing gene expression at multiple levels [248]. Furthermore, chemical modifications of RNA have emerged as a hotspot in recent years, with N6‐methyladenosine (m^6^A) modification being the most extensively studied. Added by methyltransferase complexes, m^6^A is recognized by reader proteins and affects mRNA stability or translational efficiency [249]. Studies reveal that during chronic inflammatory pain, *METTL3* expression is downregulated in the spinal cord, reducing the stability of certain anti‐inflammatory gene mRNAs and exacerbating hypersensitivity [250]; conversely, in nerve injury models, METTL14 is upregulated in DRG neurons, promoting the expression of chemokines like CCL2 via enhanced m^6^A modification, and inhibition of METTL14 alleviates neuropathic pain [251]. Thus, an imbalance in m^6^A modification can disrupt the gene network of nociceptive mediators. In summary, epigenetic and posttranscriptional modifications confer plasticity and reversibility to gene expression, opening new avenues for disease modification and sustained analgesia in chronic pain [252]. For instance, using epigenetic drugs to erase pain memory or employing gene therapy to specifically silence pain pathway genes has yielded promising results in animal models [253, 254]. If these technologies can be safely translated to the clinic to achieve a “reset” of molecular pain memory, a curative goal may be attainable.

### 3.5 Neurobiological Basis of Psychosocial Factors

#### 3.5.1 HPA Axis and Emotion–Cognition Circuits

Chronic pain and psychological stress are frequently comorbid. Chronic stress modulates nociception via the hypothalamic–pituitary–adrenal (HPA) axis: sustained stress can disrupt cortisol rhythms and increase sympathetic tone, thereby potentiating peripheral inflammatory responses and diminishing the efficacy of endogenous analgesic systems [255]. Clinical observations indicate that chronic pain patients with comorbid depression or anxiety disorders often experience more intense pain that is refractory to treatment. This is attributed to the fact that in depressive states, the activity of cerebral analgesic pathways, such as the prefrontal–midbrain–spinal circuit, is reduced, while the emotional amplification of pain is enhanced [256]. The amygdala and hippocampus play central roles in stress responses and pain memory: chronic stress can induce amygdalar hyperexcitability and hippocampal atrophy; the resulting imbalance weakens pain inhibition and consolidates fear memories associated with noxious stimuli [257]. Elevated glucocorticoid levels resulting from HPA axis overactivation can themselves exacerbate peripheral inflammation and immune dysregulation, which in turn aggravates nociceptive signaling [258]. Furthermore, chronic pain is intimately linked to sleep disturbances: sleep deprivation elevates proinflammatory cytokine levels and lowers pain thresholds, while pain itself disrupts sleep, establishing a vicious cycle [259, 260]. CBTs aimed at regulating sleep–wake rhythms have been proven to significantly improve symptoms in some chronic pain patients, underscoring the importance of the interplay between psychological and physiological factors.

A substantial component of the pain experience derives from the brain's subjective construction of pain, involving processes of expectation and learning [261]. Placebo analgesia serves as a quintessential example: when a patient anticipates that an inert treatment will alleviate pain, the brain's endogenous analgesic system is activated, resulting in a genuine reduction in pain. Imaging studies demonstrate that during the placebo response, regulation of descending pathways by the prefrontal cortex and cingulate gyrus is strengthened, accompanied by increased release within the endogenous μ‐opioid system [262]. Conversely, in the nocebo effect, negative expectations exacerbate pain; the brain generates an anxiety response with enhanced amygdalar activity and releases proalgesic CCK, which antagonizes endogenous opioids [263]. This explains why negative suggestions can intensify a patient's pain experience. If actual pain is lower than expected, the brain downregulates future pain predictions, accumulating a placebo effect; conversely, if pain exceeds expectations, fear predictions are reinforced. In the long term, if patients repeatedly experience uncontrollable pain, a cognitive pattern of “pain uncontrollability” may develop, intertwining somatic pain with learned helplessness and depressive affect. Consequently, psychological interventions aim to disrupt this negative predictive cycle; through reappraisal and the enhancement of self‐efficacy, patients can cultivate more positive cognitions and emotions regarding pain, thereby improving symptoms [78]. Mechanistically, this involves enhanced regulation of the limbic system by the prefrontal cortex, reducing the amplification of nociceptive signals.

#### 3.5.2 Systemic Interactions

Patients with chronic pain frequently exhibit an imbalance between sympathetic and parasympathetic nervous systems, characterized by low vagal tone and sympathetic predominance [264]. This is not merely a consequence of the stress response but also reciprocally influences pain [265]. Noradrenaline released by the sympathetic nervous system can act on receptors located on sensory nerve terminals and immune cells, leading to “sympathetically maintained pain” [266]. The vagus nerve, conversely, mediates the cholinergic anti‐inflammatory pathway: it modulates macrophages to release anti‐inflammatory cytokines such as IL‐10 via α7 nicotinic receptors, thereby reducing inflammation [267]. In chronic pain patients, insufficient vagal activity compromises this “braking” mechanism, facilitating the maintenance of a proinflammatory state [268]. In light of this, autonomic‐targeted therapies are being explored; for instance, vagus nerve stimulation (VNS) or transcutaneous auricular VNS aims to enhance parasympathetic input to suppress inflammation and alleviate pain [269]. Preliminary clinical studies suggest that VNS has certain efficacy in inflammatory pain conditions such as rheumatoid arthritis and can also improve comorbid depressive symptoms; however, its application in common chronic pain conditions remains under investigation [270].

The link between the gut microbiota and chronic pain represents an emerging frontier. Dysbiosis can influence host metabolism and immunity, thereby modulating nociception [271]. For instance, specific alterations in gut microbiota composition have been identified in patients with fibromyalgia and correlate with symptom severity [272]. Animal experiments indicate that germ‐free mice exhibit different sensitivities to inflammatory pain stimuli compared with mice colonized with normal flora, suggesting that microbial metabolites may influence pain perception via the gut–immune–neuro pathway [273]. Short‐chain fatty acids, key metabolites produced by the microbiota, can cross the intestinal barrier into the circulation and modulate central glial cell function [274]. Research has shown that probiotic administration can reduce hyperalgesia in mice with nerve injury, accompanied by decreased markers of spinal microglial activation [275]. Although human studies remain insufficient, this field implies that interventions such as dietary modification, probiotic supplementation, or the administration of specific metabolites hold promise as adjunctive strategies for chronic pain management [276].

Chronic pain is not solely a biological phenomenon but also a psychosocial one. Social determinants such as adverse childhood experiences, lack of social support, and financial strain have been demonstrated to correlate with the risk of developing chronic pain [277]. These factors may act upon the neuro–immune system through mediators like chronic stress and depression [278]. At the epigenetic level, childhood adversity can lead to altered methylation of HPA axis‐related gene promoters, such as NR3C1, affecting stress response sensitivity and predisposing individuals to pain‐related disorders in adulthood [279]. Social isolation is another critical variable: animal studies show that mice subjected to long‐term single housing exhibit lowered pain thresholds and higher levels of microglial activation [280]. In humans, social isolation or loneliness is associated with elevated concentrations of circulating proinflammatory cytokines like IL‐6 and CRP, as well as increased pain complaints [281]. Furthermore, cultural beliefs and social cognition shape the expression and perception of pain—certain cultures may encourage stoicism, where patients report lower pain scores despite exhibiting high internal stress hormone levels [282]. These findings underscore the necessity for an integrated BPS management approach to chronic pain, suggesting that optimal outcomes are achieved by addressing social support, psychological counseling, and lifestyle factors in concert [283].

### 3.6 Translational Bridges and Biomarkers

The translation from fundamental mechanisms to clinical application necessitates reliable translational models and detection methodologies. A significant breakthrough in this domain is the human induced pluripotent stem cell (iPSC) model: by reprogramming patient dermal fibroblasts into iPSCs and directing their differentiation into sensory neurons, scientists can “recapitulate” the patient's neuropathological characteristics in vitro [284]. For instance, drug screening using iPSC‐derived neurons from patients with congenital insensitivity to pain successfully identified methods to enhance Nav1.7 function [285]. Similarly, organoids and neuron–glia coculture systems are employed to simulate human DRG or cortical networks, facilitating the observation of interactions between distinct cell types under pain conditions [286]. Concurrently, the application of single‐cell transcriptomics and spatial omics technologies to pain‐relevant tissues in both animals and humans has assisted in mapping the “pain tissue cell atlas” [287]. These technologies enable fine discrimination of gene expression changes across different subtypes of neurons, glia, and immune cells, thereby establishing a cross‐species, cross‐stage chain of evidence [288]. For example, spatial transcriptomic analysis revealed the selective loss or downregulation of a specific subtype of spinal inhibitory neurons in chronic pain models, a finding consistent with observations in human donor spinal tissues [289]. Furthermore, advancements in in vivo imaging and interventional techniques have greatly accelerated mechanistic validation: using calcium indicators such as GCaMP6 to observe real‐time Ca^2^^+^ activity in DRG or spinal neurons of awake animals allows for the direct correlation of specific cellular discharge patterns with behavioral nociception [290]. Optogenetics and chemogenetics permit researchers to activate or silence specific classes of neurons or glia on demand, verifying their causal roles in pain behavior [291]. For instance, optogenetic activation of spinal Pdyn‐positive inhibitory neurons suppresses pain behavior, whereas silencing the same cells induces hypersensitivity; such “closed‐loop” experiments provide conceptual frameworks for therapeutic approaches like precise neuromodulation [292].

The lack of objective diagnostic indicators for chronic pain represents a major clinical bottleneck; recently, multidisciplinary collaborations have actively sought quantifiable biomarkers of pain [293]. Peripherally, intraepidermal nerve fiber density (IENFD) assessment, which counts small nerve fibers via skin biopsy, is utilized to diagnose small fiber neuropathy and fibromyalgia [294]. Many chronic pain patients exhibit reduced IENFD, suggesting peripheral nerve degeneration, which can serve as objective evidence of pain pathology [295]. Corneal confocal microscopy offers a noninvasive means to observe corneal nerve fibers, providing a window into systemic small fiber status [296]. Regarding sensory function assessment, quantitative sensory testing (QST) and laser‐evoked potentials (LEPs) are employed to map patients' sensory profiles: QST parameters, such as thermal pain thresholds, help distinguish between hyperalgesic and sensory loss phenotypes, while LEPs assess the integrity of nociceptive pathways through objective EEG responses [297]. For instance, QST demonstrating cold hyperalgesia suggests C‐fiber facilitation, whereas elevated pinprick thresholds indicate compromised Aδ‐fiber function, aiding in treatment selection [298]. In terms of fluid biomarkers, cytokines and nerve injury factors in blood and cerebrospinal fluid are elevated in certain chronic pain conditions, though their diagnostic value is limited by significant interindividual variability [293]. Interestingly, miRNA profiles in circulating exosomes may exhibit specific patterns across different pain syndromes: one study found that serum exosomal miR‐21/146a/155 levels were significantly higher in fibromyalgia patients than in controls, showing promise as adjunctive diagnostic indicators [299]. Regarding neuroimaging biomarkers, the aforementioned TSPO–PET for detecting glial activation, fMRI for extracting functional connectivity features, and MR spectroscopy for measuring neurochemical concentrations are transitioning from research to clinical translation [300]. It must be emphasized that single biomarkers are often insufficiently specific or sensitive; the future likely lies in a multimarker panel approach, integrating multiple objective datasets with clinical assessment to construct a multidimensional pain “fingerprint” for diagnostic phenotyping and therapeutic monitoring [182].

The genesis and progression of chronic pain result from the concerted action of multifactorial and multilevel processes. Peripheral nociceptor sensitization, spinal dorsal horn disinhibition and synaptic plasticity, positive feedback amplification within neuro–immune–metabolic networks, and the intervention of psychosocial factors collectively constitute an intertwined, nested maintenance loop, enabling pain to persist long‐term even in the absence of sustained peripheral injury. Encouragingly, a deeper understanding of these mechanisms is continuously spawning novel intervention strategies: from targeted drugs at the genetic and molecular levels to neuromodulation and psychotherapies at the cellular and network levels, the therapeutic armamentarium is expanding across all strata. The mechanistic research reviewed in this section provides the rationale and direction for these therapies. For instance, patients with prominent central sensitization may be more suitable candidates for drugs or techniques that enhance inhibitory pathways, while those with significant immune involvement might benefit more from anti‐inflammatory and immunomodulatory therapies. The subsequent section (Section 4) will evaluate the efficacy and evidence levels of various chronic pain management modalities based on evidence‐based medicine. Building upon the mechanism–therapy mapping discussed herein, it will propose recommendations for mechanism‐stratified, personalized analgesic regimens to guide clinical treatment. By tightly integrating mechanistic research with clinical practice, we aspire to more effectively disrupt the maintenance loops of chronic pain, achieving precise pain management that is “tailored to the symptom and the individual.”

## 4 Clinical Management Strategies for Chronic Pain

The genesis and persistence of chronic pain involve multifaceted factors across biological, psychological, and social dimensions; consequently, optimal pain management necessitates a multimodal, interdisciplinary, and patient‐centered personalized strategy [283]. Currently, a plethora of modalities are employed in clinical practice for chronic pain treatment, including pharmacotherapy, physiotherapy, psychological support, and various interventional techniques. However, the efficacy of these therapies often varies individually, and single modalities rarely achieve sufficient relief for all patients, with many carrying potential side effects or limitations [301]. Therefore, clinicians typically adopt a comprehensive treatment approach, leveraging multidisciplinary team collaboration to formulate personalized pain management plans that maximize analgesia and functional improvement [302]. This section provides a systematic review of the principal methods and recent advances in contemporary chronic pain management.

### 4.1 Pharmacotherapy

Pharmacotherapy constitutes a cornerstone of chronic pain management, encompassing nonopioid analgesics, opioids, and a variety of adjuvant analgesics. Table 2 summarizes the mechanisms of action, indications, adverse effects, and clinical recommendations for the major drug classes.

**TABLE 2:** Pharmacological management of chronic pain: major drug classes, mechanisms, and evidence‐based clinical roles.

Drug class	Representative agents	Mechanism of action	Typical indications/pain types	Key adverse effects/limitations	Clinical positioning and recommendations	References
Nonsteroidal anti‐inflammatory drugs (NSAIDs)	Oral: ibuprofen, naproxen, diclofenac; selective COX‐2: celecoxib, etc.	Inhibit COX‐1/COX‐2, reducing prostaglandin synthesis to exert anti‐inflammatory and analgesic effects	OA, rheumatoid arthritis, chronic musculoskeletal pain, some cases of chronic low back pain	Gastrointestinal bleeding/ulceration, renal impairment, increased cardiovascular risk, poor tolerability in the elderly	First‐line foundation for inflammatory pain; use “lowest effective dose for shortest duration”; assess cardiovascular and GI risks; coprescribe PPIs for high‐risk groups if necessary	[303, 304, 305]
Paracetamol (acetaminophen)	Paracetamol (acetaminophen)	Inhibits prostaglandins primarily centrally; antipyretic and analgesic, with weak anti‐inflammatory effects	Mild‐to‐moderate osteoarthritis, chronic low back pain, headache, etc.; particularly for those with NSAID contraindications	Increased risk with alcohol or other hepatotoxic drugs	Combine with NSAIDs to reduce their dosage; not suitable as sole long‐term therapy for moderate‐to‐severe chronic pain	[306, 307, 308]
Opioid analgesics	Morphine, oxycodone, hydromorphone, tramadol, fentanyl patches, etc.	Activates μ‐opioid receptors, inhibiting nociceptive transmission and enhancing descending inhibition	Moderate‐to‐severe cancer pain; select cases of severe chronic noncancer pain	Constipation, somnolence, cognitive impairment, respiratory depression, endocrine disruption; risk of misuse and addiction	Not first‐line for chronic noncancer pain; consider only short‐term, low‐dose adjunctive use when other therapies fail and benefits outweigh risks; strict monitoring and periodic tapering assessment required	[309, 310, 311, 312, 313]
Antidepressants (TCAs/SNRIs, etc.)	TCAs: amitriptyline, nortriptyline; SNRIs: duloxetine, milnacipran, etc.	Increase 5‐HT and noradrenaline levels in descending inhibitory pathways; some also modulate ion channels	Neuropathic pain (DPN, PHN), fibromyalgia, tension‐type headache prophylaxis, chronic pain comorbid with depression/anxiety	TCAs: dry mouth, constipation, weight gain, arrhythmia risk; SNRIs: nausea, insomnia, elevated BP; highly variable individual tolerance	Key “adjuvant analgesics” for neuropathic pain and fibromyalgia, especially with comorbid mood/sleep disorders; start low, titrate slowly, and assess cardiovascular risks	[314, 315, 316, 317, 319]
Gabapentinoid anticonvulsants (α2δ ligands)	Gabapentin, pregabalin	Bind to α2δ subunit of voltage‐gated calcium channels, reducing excitatory transmitter release and inhibiting central sensitization	Various neuropathic pain conditions (PHN, DPN, SCI‐related pain, etc.), fibromyalgia	Dizziness, somnolence, edema, weight gain, cognitive decline; increased risk of respiratory depression/death when combined with opioids/benzodiazepines; potential for misuse and dependence	First/second‐line for neuropathic pain; not recommended for widespread chronic pain lacking clear neuropathic components; monitor for sedation, falls, and misuse risk	[320, 321, 322, 323, 324, 325, 326, 328]
CGRP pathway agents	Anti‐CGRP/receptor mAbs: erenumab, fremanezumab, galcanezumab, etc.; “gepants”: ubrogepant, rimegepant, etc.	Block CGRP peptide or receptor, inhibiting nociceptive transmission in the trigeminal system	Preventative and acute treatment for migraine; efficacy for cluster headache and other craniofacial pain is under investigation for some agents	Injection site reactions, constipation, mild infections; long‐term cardiovascular safety under surveillance; high cost	Crucial option for refractory moderate‐to‐severe migraine; recommend individualized use after weighing cost‐effectiveness against traditional preventatives	[329, 330, 331, 332, 333, 335, 336]
Anti‐NGF monoclonal antibodies	Tanezumab, fasinumab, etc.	Neutralize NGF, blocking its binding to TrkA receptors, thereby reducing nociceptor sensitization and aberrant sprouting	Moderate‐to‐severe OA pain, chronic low back pain, etc.	Associated with rapidly progressive osteoarthritis (RPOA) and increased joint replacement rates; risk correlates closely with dosage and concurrent NSAID use	Not routinely approved; considered only in trials or strictly selected patients; reflects disease‐modifying analgesia concept, but joint safety concerns remain unresolved	[337, 338, 339, 340]
Sodium channel/other ion channel modulators	Selective Nav1.7/Nav1.8 blockers, P2×3 antagonists, TRPV1/ASIC antagonists, etc.	Target sensory neuron‐specific ion channels, reducing action potential generation or conduction to diminish nociceptive input	Acute postoperative pain, neuropathic pain, chronic cough‐related pain, etc.	Adverse effects vary by target,, dysgeusia (P2×3 antagonists), hyperthermia or thermoregulatory issues (TRPV1 antagonists); extremely high demands on selectivity and safety window	Represents the frontier of nonopioid analgesia, mostly in clinical trials or early market stages; promises more precise targeted therapy for specific pain phenotypes in the future	[342, 343, 344, 345, 346, 347, 348, 350]

#### 4.1.1 Nonopioid Analgesics

Nonsteroidal anti‐inflammatory drugs (NSAIDs) and paracetamol (acetaminophen) serve as first‐line pharmacological options for the relief of mild‐to‐moderate chronic pain. NSAIDs exert analgesic and anti‐inflammatory effects by inhibiting cyclooxygenase (COX) enzymes, thereby reducing prostaglandin synthesis [303]. They demonstrate efficacy in inflammatory conditions such as OA, rheumatoid arthritis, and chronic musculoskeletal pain, often being utilized in combination with other therapies [304]. Regarding safety, long‐term NSAID use is associated with gastric mucosal injury, gastrointestinal ulceration and bleeding, as well as an increased risk of cardiovascular events and renal impairment. Recent evidence indicates that selective COX‐2 inhibitors do not significantly increase the risk of myocardial infarction in high‐risk cardiovascular patients and offer better gastrointestinal tolerability; however, they should still be prescribed at the lowest effective dose for the shortest duration to minimize adverse events [305]. Paracetamol, exerting antipyretic and analgesic effects via central inhibition of prostaglandins and other mechanisms, lacks significant anti‐inflammatory activity but is widely used for basal analgesia across various chronic pain conditions due to its favorable safety profile [306]. It is commonly indicated for OA, low back pain, and headache, particularly in patients with contraindications to NSAIDs [307]. At recommended doses, paracetamol has few side effects, though overdose can cause hepatotoxicity. Notably, recent systematic reviews suggest that paracetamol monotherapy offers limited analgesic benefit for long‐term control of chronic OA or low back pain. Consequently, clinicians frequently combine paracetamol with NSAIDs to achieve synergistic analgesia and allow for NSAID dose reduction [308]. In summary, nonopioid analgesics remain the preferred pharmacological intervention for chronic pain due to their relatively manageable side effect profile, yet their use requires a careful balance of efficacy against long‐term risks, with individualized selection and periodic safety assessments.

#### 4.1.2 Opioid Analgesics

Opioids produce potent analgesia by activating central and peripheral μ‐opioid receptors and represent a critical therapeutic modality for moderate‐to‐severe chronic pain, particularly cancer pain [309]. Common agents include morphine, oxycodone, and fentanyl, which offer the advantage of providing significant relief for intense pain. However, the long‐term application of opioids carries substantial risks: they are associated with common adverse effects such as constipation, somnolence, and cognitive dysfunction, and more gravely, with the development of tolerance, physical dependence, and addiction [310]. It is estimated that approximately 3–12% of chronic pain patients treated with opioids may develop misuse or addiction tendencies [311]. Against the backdrop of the current opioid crisis, the use of opioids for chronic noncancer pain demands extreme caution. The 2022 US CDC guidelines emphasize that opioids should not be considered first‐line therapy for chronic pain unless other treatments have failed and the expected benefits outweigh the risks [312]. If their use is deemed necessary, therapy should be initiated at the lowest effective dose, with close follow‐up of efficacy and side effects, and periodic assessment for tapering or discontinuation. Research indicates that long‐term high‐dose opioid therapy is associated with severe harm, with risks increasing in a dose‐dependent manner. Consequently, guidelines recommend incorporating opioids into multimodal analgesic regimens, combined with nonopioid medications and nonpharmacological therapies, to minimize opioid consumption [312]. Additionally, research indicates that there are significant sexual dimorphisms in the pharmacological properties of opioids: compared with men, women typically require lower doses of morphine to achieve equivalent analgesic effects, but report a significantly higher incidence of adverse reactions such as nausea, vomiting, and respiratory depression. Conversely, men may exhibit a slower onset of analgesia but better tolerability profiles [313]. Regarding the rational use of opioids, several strategies have been developed in recent years: employing extended‐release formulations to stabilize plasma concentrations, selecting partial agonists or mixed agonist‐antagonists to reduce addiction risk, and strengthening patient–provider agreements and monitoring to prevent misuse. In conclusion, opioid indications in chronic pain management must be strictly selected, adhering to the principle of “avoid if possible, minimize if necessary,” and closely monitored in accordance with the latest guidelines to balance analgesic benefits against risks [314].

#### 4.1.3 Adjuvant Analgesics

Adjuvant analgesics refer to a class of medications not originally designed for pain relief but which demonstrate efficacy in alleviating certain chronic pain conditions; they primarily include antidepressants and anticonvulsants. These agents play a particularly significant role in the management of neuropathic pain and syndromes such as fibromyalgia. At the same time, they not only alleviate the corresponding pain symptoms but also exert favorable effects on concomitant symptoms. Gabapentinoids (pregabalin) are particularly advantageous for patients with comorbid insomnia, as they can reduce sleep latency and increase slow‐wave sleep while dampening neuronal excitability [315]. In contrast, 5‐HT–noradrenaline reuptake inhibitors (duloxetine) and tricyclic antidepressants (TCAs) are more suitable for patients with “pain–depression” comorbidity, simultaneously targeting nociception and affective dysregulation through shared monoaminergic mechanisms. Prioritizing these agents in patients with multiple symptoms not only simplifies medication regimens but also reduces the risk of polypharmacy interactions.

Specific antidepressants, particularly SNRIs and TCAs, have been proven to possess adjuvant analgesic properties in chronic pain [316]. Their primary mechanism involves increasing the levels of 5‐HT and noradrenaline in central descending inhibitory pathways, thereby enhancing the suppression of nociceptive signals [317]. Clinically commonly used agents include duloxetine and milnacipran (SNRIs), as well as amitriptyline and nortriptyline (TCAs), all of which demonstrate efficacy in conditions such as diabetic peripheral neuropathy, postherpetic neuralgia, fibromyalgia, and the prophylaxis of tension‐type headache [318]. A recent meta‐analysis revealed that duloxetine significantly reduces pain in patients with fibromyalgia [319]; however, antidepressants generally provide only modest improvement for nonfibromyalgia musculoskeletal pain and are prone to discontinuation due to side effects. Common adverse effects include nausea, insomnia, and elevated blood pressure with SNRIs, and dry mouth, constipation, somnolence, and cardiac conduction block with TCAs due to their anticholinergic properties, necessitating gradual titration from low doses. Nevertheless, for chronic pain patients with comorbid depression or anxiety, these medications offer the advantage of “dual efficacy,” simultaneously improving mood and sleep, thereby indirectly enhancing pain tolerance. It is noteworthy that guidelines vary regarding the use of antidepressants for chronic pain: the American College of Physicians guidelines suggest duloxetine as a second‐line option for CLBP when nonpharmacological treatments fail, whereas the National Institute for Health and Care Excellence guidelines are relatively more conservative regarding its use in this condition [320]. On the other hand, studies have shown that due to sex differences in descending modulation pathways and transporter availability, women with neuropathic pain may respond more favorably to serotonergic agents, whereas men may derive greater benefit from tricyclic agents, though these associations remain under further investigation [321]. In summary, antidepressants play a crucial adjuvant role in chronic pain management, particularly for neuropathic pain or patients with affective disorders; however, their selection should be judicious, weighing benefits against side effects and incorporating patient preference [322].

Gabapentinoid anticonvulsants exert analgesic effects by modulating the α2δ subunit of voltage‐gated calcium channels, thereby reducing central sensitization and neuronal excitability [323]. They are primarily indicated for various neuropathic pain conditions, including postherpetic neuralgia, diabetic peripheral neuropathy, and central neuropathic pain following spinal cord injury, as well as for fibromyalgia [324]. Pregabalin is approved for postherpetic neuralgia and fibromyalgia, demonstrating utility in pain reduction and sleep improvement [325]. For instance, in patients with postherpetic neuralgia, the number needed to treat (NNT) for gabapentin is approximately 6.7. However, anticonvulsants are not effective for all chronic pain conditions: a randomized controlled trial in CLBP showed no significant difference in efficacy between gabapentin and placebo [326]. Furthermore, safety concerns regarding gabapentinoids have emerged in recent years. On one hand, common side effects include sedation, dizziness, edema, and cognitive decline [327]; on the other hand, their potential for misuse and dependence is increasingly recognized, particularly when coprescribed with opioids or benzodiazepines, which may elevate the risk of respiratory depression and mortality [328]. Research has found that mortality rates in patients using gabapentinoids are significantly higher than in the general population [329]. Consequently, current research advises against the use of gabapentin and pregabalin for broad chronic pain indications lacking a definitive neuropathic diagnosis. In summary, while anticonvulsants are supported by robust evidence and can provide significant analgesia in classic neuropathic pain [330], their efficacy in non‐neuropathic chronic pain is limited. Their use requires a careful weighing of benefits against potential harms, positioning them as a component of a multimodal regimen rather than a first‐line option [331].

#### 4.1.4 Emerging Pharmacotherapies

As the understanding of chronic pain mechanisms deepens, progress has been made in recent years regarding novel drug targets and corresponding therapies. Representative directions include the CGRP pathway, the NGF pathway, and modulators of specific ion channels.

CGRP is a neuropeptide playing a critical role in migraine and other pain conditions, capable of inducing vasodilation and facilitating nociceptive transmission. In the past 5 years, breakthrough progress has been achieved in therapeutics targeting the CGRP pathway, with a series of monoclonal antibodies and small‐molecule antagonists approved for migraine prevention and acute treatment [332]. For instance, anti‐CGRP receptor antibodies such as erenumab, as well as “gepant” class small molecules like ubrogepant and rimegepant, have all been proven effective in reducing migraine frequency or attenuating pain intensity during attacks [333, 334, 335, 336]. Compared with traditional migraine medications, CGRP‐targeted therapies exhibit fewer vascular side effects and are less likely to induce medication‐overuse headache, significantly improving QoL for patients with refractory migraine [337]. The success of CGRP antagonists has also inspired new avenues in other pain domains: while their primary indication remains migraine, the role of CGRP in conditions such as cluster headache and trigeminal neuralgia is currently under investigation [338]. Preliminary studies are exploring the use of CGRP monoclonal antibodies for refractory facial pain, though efficacy remains to be established. Furthermore, given the physiological function of CGRP in the cardiovascular system, the use of CGRP antagonists requires careful assessment in patients with cardiovascular risk factors [339]. Overall, the emergence of CGRP pathway drugs marks a new chapter in targeted pain pharmacotherapy, providing a crucial novel nonopioid analgesic option for patients with intractable headaches.

NGF plays a significant role in the generation and maintenance of pain. Studies have found that in chronic pain states such as OA, NGF levels are elevated and correlate with pain severity. Monoclonal antibodies targeting NGF, representing a novel class of analgesics with a distinct mechanism of action, have shown promising efficacy in recent clinical trials for OA pain and CLBP [340]. A Phase III clinical study confirmed that anti‐NGF antibodies significantly reduce pain and improve function in patients with moderate‐to‐severe OA [341]. However, safety concerns regarding NGF inhibitors have hindered their clinical prospects. Most notably, a subset of patients developed rapidly progressive OA (RPOA), characterized by a drastic narrowing of the joint space and accelerated bone destruction within a short period [342]. Although the overall rate of serious adverse events did not differ substantially, concerns over joint safety have led the United States Food and Drug Administration (US FDA) to withhold approval for these agents to date. Researchers are attempting to mitigate risks by lowering doses and rigorously selecting patients, such as avoiding concurrent use with NSAIDs [343]. Some experts posit that NGF inhibitors may hold specific value for OA patients who are immobilized by pain and refractory to other therapies, provided they are used under careful monitoring [344]. In summary, anti‐NGF therapy reflects a paradigm shift in pain management from general analgesia to disease‐modifying analgesia—alleviating pain at its source by blocking algesic factors. Nevertheless, its clinical application will remain strictly limited until safety concerns are resolved.

Peripheral and central ion channels play pivotal roles in nociceptive signal transduction, making them hotspots for recent drug development. Most notably, research has focused on voltage‐gated sodium channels (Nav subtypes) as analgesic targets [345]. Nav1.7, Nav1.8, and Nav1.9 are sodium channel subtypes primarily expressed in sensory neurons and hold significant positions in pain genesis [346]. In particular, Nav1.7 acts as a “threshold switch” for action potential generation and is linked to various human hereditary pain disorders: loss‐of‐function mutations in Nav1.7 result in congenital insensitivity to pain, whereas gain‐of‐function mutations cause intractable pain syndromes [108]. Nav1.8 primarily mediates peripheral transmission of inflammatory and neuropathic pain signals, while Nav1.9 is implicated in cold nociception and small fiber neuropathy [347]. Based on these findings, scientists have dedicated substantial effort to developing biotoxin derivatives or small molecules to selectively block these channels, aiming to mimic the effects of congenital analgesia [348]. To date, multiple Nav1.7 inhibitors have entered clinical trials; for instance, selective Nav1.7 blockers such as PF‐05089771 and BIIB074 (vixotrigine) have undergone Phase II trials in populations with painful diabetic peripheral neuropathy and trigeminal neuralgia [122, 349]. Concurrently, the broad‐spectrum sodium channel inhibitor ANP‐230, which blocks Nav1.7/1.8/1.9, has been reported to possess clinical development potential and demonstrated superior analgesic effects compared with existing drugs in animal models [350]. Animal model results are encouraging: for example, the novel candidate ANP‐230 showed better analgesic efficacy than pregabalin across various pain models [350]. However, successfully translating ion channel blockers into clinical analgesics remains a formidable challenge. Most Nav inhibitors have failed to meet efficacy endpoints in clinical trials due to insufficient selectivity, dose limitations, and safety issues [351]. For instance, Nav1.7 inhibitors may have limited efficacy due to difficulty in blocking a sufficient proportion of neurons simultaneously, and side effects emerge at high doses. Addressing these issues, researchers are exploring new strategies, such as multitarget combinatorial inhibition or improved delivery routes to enhance targeting [352]. Beyond sodium channels, other ion channels like TRPV1, ASICs, and P2×3 are also under development: TRPV1 antagonists previously showed good analgesia but were hampered by side effects such as hyperthermia; P2×3 receptor antagonists have recently been successfully employed for chronic cough and are being trialed for neuropathic pain relief [353]. In summary, novel ion channel modulators represent a frontier in nonopioid analgesic drug development. If technical hurdles can be overcome, they hold the promise of delivering revolutionary therapies for intractable chronic pain.

### 4.2 Nonpharmacological Therapies

Nonpharmacological therapies constitute a vital component of comprehensive chronic pain management, aligning particularly well with the BPS model of chronic pain [283]. These modalities encompass physical agent modalities, psychological/behavioral interventions, patient education, and various complementary therapies. Their objective is to improve patients' physical function, modulate pain cognition and emotional responses, and thereby globally reduce the impact of pain on daily life [354, 355].

#### 4.2.1 Physiotherapy

Regular exercise therapy is central to the long‐term management of chronic pain. Exercise regimens, including aerobic exercise, resistance training, and flexibility exercises, reduce pain‐related disability and enhance physical mobility and QoL [356]. Exercise also promotes endogenous analgesic mechanisms, attenuates inflammation and muscle tension, and aids in alleviating the depression and sleep disturbances frequently comorbid with chronic pain [357]. Transcutaneous electrical nerve stimulation (TENS) is another commonly employed physical modality, involving the application of low‐intensity electrical current via cutaneous electrodes to stimulate sensory nerves and block nociceptive transmission [358]. TENS is convenient, has minimal side effects, and can be utilized as a home‐based analgesic strategy for various conditions such as low back pain and arthralgia. Although clinical trial results for TENS are variable, numerous patients subjectively report temporary pain relief, particularly when combined with exercise and physiotherapy [359]. Acupuncture, a hallmark of traditional Chinese medicine, is now accepted in the West as a complementary therapy for chronic pain. Acupuncture induces analgesia by stimulating specific acupoints to trigger endogenous opioid peptide release and neuromodulation [360]. Extensive randomized controlled trials and meta‐analyses indicate that acupuncture provides small‐to‐moderate pain reduction compared with placebo (sham acupuncture) [361]. While part of the analgesic effect of acupuncture derives from the placebo effect, its overall benefit is recognized; consequently, some studies recommend acupuncture as an adjunctive option for certain primary chronic pain conditions [362]. Physiotherapy also includes thermotherapy/cryotherapy and massage, which help patients relax and temporarily diminish nociceptive signaling [363, 364]. It must be emphasized that the effectiveness of physiotherapy often hinges on patient adherence and compliance. Therapists should design comprehensive plans tailored to the patient's condition—integrating exercise with physical modalities and home practice—and conduct regular follow‐ups to adjust the regimen for maximal efficacy.

#### 4.2.2 Psychological and Behavioral Therapies

Chronic pain is frequently accompanied by significant psychological distress and behavioral changes, making psychological interventions pivotal in pain management. CBT is the most widely applied psychological therapy for pain. It educates patients on the cognitive–emotional impact of pain and teaches relaxation training and coping skills to alter negative thought patterns and behaviors associated with pain [365]. CBT has been proven effective in reducing depression and anxiety levels in chronic pain patients, enhancing their sense of control over pain, and, to a certain extent, alleviating pain intensity and improving daily functioning [365, 366, 367]. For instance, CBT instructs patients to divert attention from pain, re‐establish positive activity schedules, and employ progressive muscle relaxation and breathing exercises to relieve muscle tension, thereby breaking the vicious cycle of “pain–insomnia–emotional deterioration–worsening pain” [368]. Acceptance and commitment therapy (ACT) is an emerging “third‐wave” psychological therapy that emphasizes accepting unavoidable pain experiences while focusing on personal values and life goals. Through mindfulness practice and the cultivation of psychological flexibility, ACT aims to reduce the suffering associated with pain [369]. Studies indicate that ACT is significantly superior to controls in enhancing pain acceptance and QoL, as well as reducing pain interference, anxiety, and depression in chronic pain patients [370]. These results suggest that ACT serves as a beneficial complement to CBT, being particularly effective in alleviating the helplessness and psychological distress arising from the incurable nature of chronic pain [371]. Biofeedback is a technique utilizing instrumentation to feedback physiological signals—such as electromyography (EMG) and skin temperature—to train patients in the autonomic regulation of bodily responses. In chronic headache and muscle pain, biofeedback helps patients become aware of and relax overly tense muscles and lower sympathetic arousal, thereby mitigating symptoms [372]. For example, patients with tension‐type headache who learn to relax frontalis and cervical muscles via EMG biofeedback experience reduced headache frequency and intensity [373]. Mindfulness‐based stress reduction (MBSR) cultivates awareness and an attitude of acceptance through meditation and mindful breathing exercises, encouraging patients to observe pain and emotions nonjudgementally to reduce suffering [374]. Research has found that chronic pain patients participating in an 8‐week MBSR course exhibit statistically significant reductions in pain intensity, depression, and insomnia scores, along with improved pain coping abilities, compared with control groups [375, 376]. The efficacy of mindfulness therapy is comparable to that of CBT, positioning it as a promising self‐management tool. Furthermore, patient education and self‐management permeate all psychological interventions—helping patients fully comprehend the mechanisms of chronic pain, establish reasonable expectations for functional recovery, and learn to utilize pharmacological and nonpharmacological modalities, thereby enhancing their willingness and confidence to actively participate in treatment [377, 378]. In conclusion, psychological and behavioral therapies, by improving patients' cognitive and emotional responses to pain and transforming them from passive sufferers to active copers, are an indispensable component of holistic chronic pain treatment [365, 378].

### 4.3 Interventional Therapies

When conventional pharmacotherapy and conservative management prove insufficient for pain control, interventional pain therapies offer further analgesic options. By targetedly blocking or modulating nociceptive signal transmission pathways, interventional therapies often provide significant relief for intractable pain in specific regions [379]. These primarily include minimally invasive procedures such as nerve blocks and radiofrequency ablation (RFA), as well as neuromodulation techniques.

#### 4.3.1 Nerve Blocks and RFA

Nerve blocks involve the precise injection of local anesthetics or corticosteroids near neural structures transmitting pain signals, aiming to temporarily interrupt neural conduction or reduce local inflammation for analgesia [380]. Common procedures include epidural blocks, dorsal ramus blocks, sympathetic nerve blocks, and peripheral nerve blocks. Nerve blocks offer rapid onset, significantly reducing pain and improving patient mobility, often buying time for subsequent rehabilitation [381]. However, their analgesic duration is limited, and some patients require repeated injections to maintain efficacy. To extend analgesia, RFA can be employed. Under X‐ray or ultrasound guidance, an RF electrode is placed near the target nerve, and high‐frequency current is applied to heat and destroy nerve conduction function [382]. RFA is categorized into continuous (thermal) and pulsed modes: thermal RFA thoroughly denervates the target, typically providing analgesia lasting from 6 months to over a year, and is suitable for indications such as ablating medial branch nerves in facet joint pain or sensory nerves in knee OA [383, 384, 385, 386]; pulsed RF acts on nerves in a gentler manner without causing tissue coagulation, suppressing conduction for months with lower risk. Taking lumbar facet joint pain as an example, thermal RF ablation of the medial branch nerves significantly reduces pain and improves function, with reported success rates around 50%[387]. Similarly, for patients with knee OA ineligible for surgery, RF ablation of knee sensory nerves demonstrated superior analgesic efficacy and functional improvement compared with controls in randomized controlled trials [386, 388]. Side effects of RFA include local numbness and dysesthesia, which are generally acceptable. Other interventional methods, such as percutaneous nucleoplasty for disc herniation and intra‐articular injections of platelet‐rich plasma or stem cells, alleviate pain through distinct mechanisms and also fall within the scope of interventional therapy [389, 390]. Overall, minimally invasive interventions like nerve blocks and RFA offer significant and safe analgesia for well‐localized chronic pain conditions, such as segmental neuralgia and arthralgia. They serve a “bridging” role in multimodal management: providing transitional pain relief to facilitate functional rehabilitation, or acting as a repeatable long‐term treatment option to reduce the need for systemic analgesics like opioids.

#### 4.3.2 Neuromodulation Technologies

In recent years, the evolution of implantable neurostimulation devices has offered renewed hope for refractory chronic pain. Spinal cord stimulation (SCS) represents one of the most established neuromodulation therapies [391]. Its underlying principle involves the implantation of electrodes into the epidural space to deliver pulsed electrical current to the dorsal columns, generating “paresthesia coverage” or modulating descending pain control pathways to inhibit the ascending transmission of nociceptive signals [152]. SCS is particularly indicated for intractable neuropathic pain conditions, such as failed back surgery syndrome, CRPS, and posttraumatic peripheral neuropathy [392]. For long‐term pain refractory to pharmacotherapy, SCS can deliver clinically significant and sustained analgesia alongside functional improvement [393]. Recent innovations in SCS technology include high‐frequency stimulation (10 kHz) which provides paresthesia‐free analgesia, dorsal root ganglion stimulation (DRG‐S) for precise targeting of focal pain, and closed‐loop feedback SCS that adjusts output based on patient posture [394, 395]. Studies indicate that closed‐loop SCS is superior to traditional open‐loop systems in terms of pain relief and functional outcomes [396]. Risks associated with SCS primarily involve implant‐related complications—such as infection, dural puncture, electrode migration, or device malfunction—though the overall incidence is low, and the therapy is considered reversible as the device can be deactivated or explanted.

Peripheral nerve stimulation (PNS) involves the electrical modulation of specific peripheral nerves or plexuses to alleviate pain within their distribution [397]. Traditional PNS requires the implantation of electrodes near the target nerve—for example, occipital nerve stimulation for refractory occipital neuralgia or migraine, and femoral or sciatic nerve stimulation for lower limb neuropathic pain [398]. Recently, 60‐day absorbable microstimulation leads have emerged, allowing for peripheral nerve modulation over a defined period without permanent implantation; these have yielded positive results in studies of chronic knee and shoulder pain [399]. A study on knee OA pain demonstrated that a 2‐month PNS treatment group showed significant improvements in pain intensity and functional disability compared with controls, with effects persisting even after stimulation cessation [400]. While PNS is suitable for localized mononeuropathies, its utility for multifocal widespread pain remains limited [401].

Deep brain stimulation (DBS) involves implanting electrodes into central nuclei associated with pain modulation—such as the thalamus or the PAG—for continuous stimulation. It is principally reserved for extremely severe, intractable pain cases where all other therapies have failed [402]. Although attempted since the 1960s, efficacy reports vary: some small‐sample studies indicate that approximately 50% of patients achieve greater than 50% pain reduction, often accompanied by mood improvement [403]. Given that DBS requires intracranial surgery and entails high costs and significant risks, its application is currently restricted to investigational use for extreme cases in select centers, with its efficacy and indications still under exploration [404]. Additionally, other minimally invasive interventions, such as intrathecal drug delivery pumps and high‐intensity focused ultrasound ablation of thalamic nuclei, also show promise in specific domains [405, 406]. In summary, neuromodulation technologies provide a new horizon for patients suffering from pain refractory to conventional therapies; however, their cost and invasiveness dictate that strict patient selection and assessment by specialized teams are essential.

Chronic pain management encompasses multiple dimensions, including pharmacotherapy, physical/rehabilitation therapy, psychological intervention, and interventional therapy. While each modality possesses distinct mechanisms of action and target populations, efficacy is highly individualized, precluding a “one‐size‐fits‐all” approach [407, 408]. Many patients require continuous adjustment and combination of multiple therapies throughout long‐term management, whilst navigating limitations such as tolerance, side effects, and complications. This reality drives the exploration of precision medicine‐oriented therapies—leveraging pain phenotyping and biomarker screening to match patients with the most appropriate therapeutic combinations, alongside developing novel analgesic targets and technologies to enhance overall efficacy [409, 410]. Practically, the most viable pathway lies in multimodal, interdisciplinary comprehensive management: aconstructing a patient‐centered “pain management wheel” that organically integrates pharmacotherapy, physiotherapy, psychological support, interventional procedures, and lifestyle modifications. This holistic approach aims to alleviate physiological pain while simultaneously addressing psychological and social functioning, thereby maximizing the overall health and QoL for patients with chronic pain.

## 5 Emerging Therapies: From Preclinical to Clinical Trials

As the understanding of pain mechanisms deepens, a plethora of innovative analgesic therapies targeting novel sites are currently under development. Over the past decades, researchers have identified numerous receptors, ion channels, and enzymes as potential targets for analgesic drugs [411]. However, progress in the clinical treatment of chronic pain remains limited; existing analgesics often fail to provide adequate relief due to insufficient efficacy or dose‐limiting side effects [412]. There is an urgent imperative to develop novel, high‐efficacy, and well‐tolerated nonopioid analgesic modalities. Given the substantial investment in chronic pain mechanism research, it was anticipated that multiple new classes of analgesics would emerge by the 2020s; yet, actual translational outcomes have been sparse [413]. This discrepancy stems from bottlenecks in translating animal research to human trials, including an incomplete understanding of pain mechanisms, inappropriate target selection, poor pharmacokinetic properties of clinical candidates, and the failure of animal models to fully recapitulate the pathobiology of human chronic pain. This section will highlight the most promising preclinical animal studies and review relevant clinical trial progress, aiming to illustrate how these emerging therapies may offer new hope for patients with chronic pain.

### 5.1 Animal Models of Chronic Pain

**FIGURE 4:**
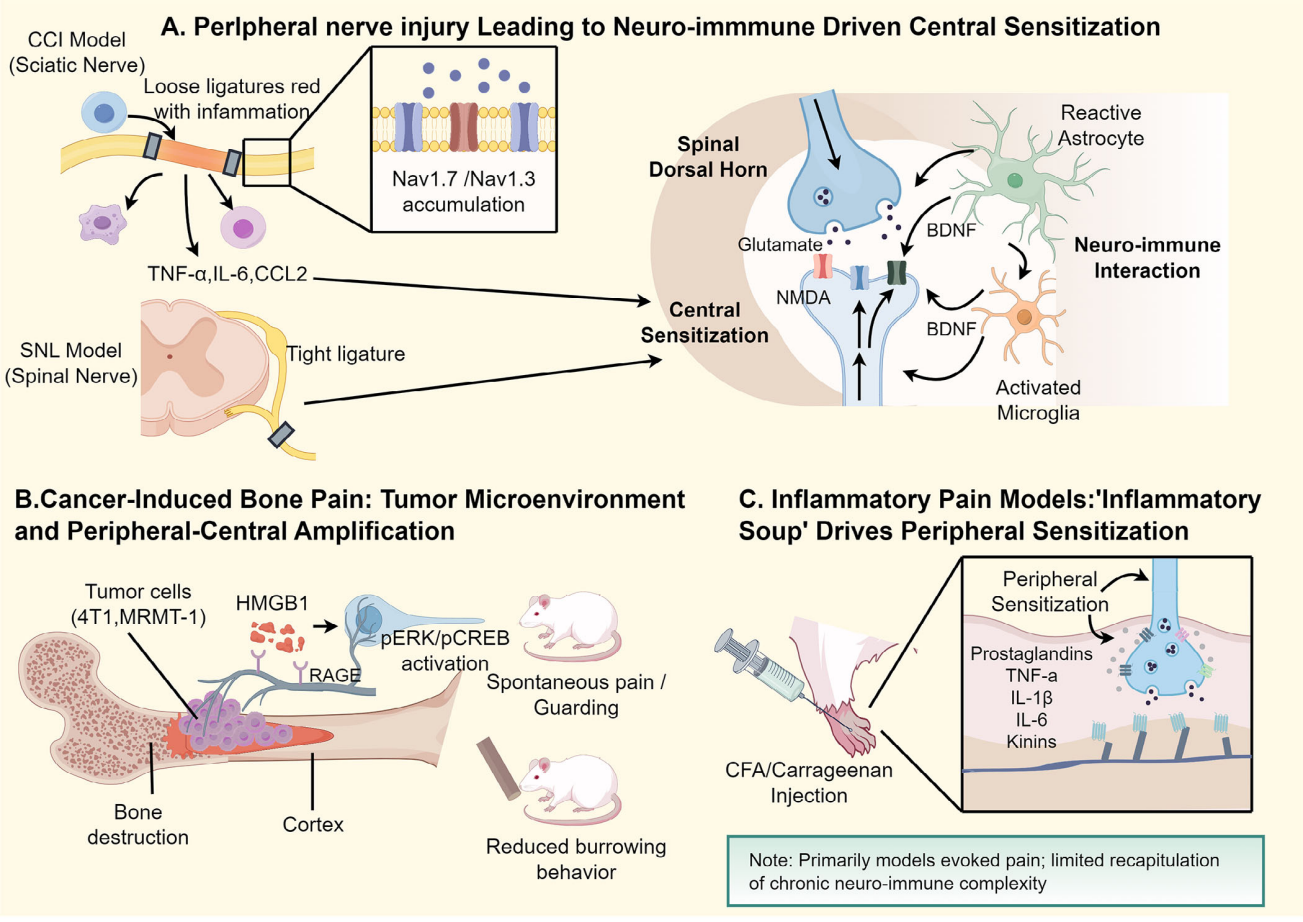
Preclinical animal models of chronic pain and key mechanisms. (A) Neuropathic pain models: peripheral nerve injury and neuro–immune‐driven central sensitization. The chronic constriction injury (CCI) model uses loose ligation of the sciatic nerve to produce focal nerve compression, local inflammation and clustering of voltage‐gated sodium channels. The spinal nerve ligation (SNL) model involves tight ligation of one or more spinal nerve roots. Injury signals from both models converge on the dorsal horn, where activation of postsynaptic NMDA receptors and neuron–glia crosstalk promotes central sensitization. Activated glial cells release mediators such as BDNF, further enhancing dorsal horn neuronal excitability. (B) Cancer‐induced bone pain (CIBP) models: tumor microenvironment‐driven peripheral‐to‐central amplification. Injection of tumor cells into the bone marrow cavity results in progressive bone destruction and the development of an acidic microenvironment. HMGB1 binds to the receptor for advanced glycation end products (RAGE) on sensory nerve fibers, engaging pERK/pCREB signaling in dorsal root ganglion (DRG) neurons. These pathological changes translate behaviorally into ongoing (spontaneous) pain and functional impairment. (C) Inflammatory pain models: “inflammatory soup” and peripheral sensitization. Local injection of complete Freund's adjuvant (CFA) or carrageenan into the hindpaw evokes robust tissue inflammation. In the magnified view, primary afferent terminals are bathed in an “inflammatory soup” rich in prostaglandins, TNF‐α, IL‐1β, IL‐6, and kinins, which act directly on nociceptor endings to increase membrane excitability and drive peripheral sensitization.

The chronic constriction injury (CCI) and spinal nerve ligation (SNL) models are commonly employed animal models of neuropathic pain, utilized to simulate chronic pain resulting from nerve injury (Figure 4) [414]. The CCI model, which involves loose ligation of the sciatic nerve to induce partial nerve compression, reliably provokes typical neuropathic pain behaviors in test animals [415]. Research using the CCI model has elucidated mechanisms relevant to human chronic pain, such as the role of peripheral neuronal sodium channels Nav1.7 and Nav1.3 in neuropathic pain. Furthermore, experiments have demonstrated that inhibiting the activity of inflammatory cytokines or chemokines in the CCI model can attenuate pain behaviors, suggesting that inflammatory mediators are potential targets for analgesic intervention [416, 417]. The SNL model induces persistent pain manifestations, such as tactile allodynia and thermal hyperalgesia, through the ligation of spinal nerves. This model has been instrumental in clarifying the mechanisms of central sensitization in persistent pain, including the involvement of NMDA receptors and BDNF in the establishment of central sensitization [418, 419]. Notably, studies utilizing the SNL model have found that inhibiting the activation of microglia and astrocytes effectively alleviates pain induced by SNL. This evidence supports the criticality of “neuro–immune” mechanisms in neuropathic pain and provides a rationale for targeting glial cells as novel analgesic sites [420, 421, 422].

Regarding cancer pain models, “cancer‐induced bone pain” (CIBP) is the most representative. A common methodology involves injecting syngeneic tumor cells into the intramedullary cavity of the tibia or femur to induce progressive bone destruction. This elicits behavioral changes such as mechanical/thermal hypersensitivity, asymmetrical weight‐bearing, and spontaneous pain, while recapitulating features of bone metastasis at radiological and histological levels [423, 424, 425, 426]. These models have elucidated pathophysiological processes involving peripheral–central cascade amplification: for instance, the MRMT‐1 model exhibits electrophysiological and molecular evidence of enhanced spinal dorsal horn neuronal excitability and central sensitization [427]. Mechanistically, an acidic microenvironment, neuro–immune interactions, and neurotrophic factors collectively contribute to the genesis and maintenance of CIBP. In the case of 4T1‐induced bone metastasis, activation of the HMGB1/RAGE axis drives pERK/pCREB signaling and axonal regeneration in the DRG; pharmacological blockade of this axis attenuates pain behaviors [428]. In terms of behavioral assessment, beyond von Frey testing and weight‐bearing/limb use scores, nonevoked metrics such as burrowing are valuable for capturing trajectories of functional impairment and spontaneous pain, demonstrating good reproducibility and translational relevance [429]. Notably, glial involvement exhibits heterogeneity across models and temporal phases: some studies suggest late‐stage microglial activation characterized by the P2×7R–p38–IL‐18 pathway, inhibition of which alleviates bone cancer pain [430]; conversely, other studies in rat CIBP models have not observed typical spinal microglial responses, indicating that cell line, host background, and disease stage significantly influence pathological readouts [431]. Beyond bone pain, models of primary tumor‐associated pain, such as oral squamous cell carcinoma, are utilized to investigate trigeminal sensitization and the sensitizing effects of tumor microenvironment algesic mediators on peripheral sensory nerves, supporting a cross‐species and cross‐hierarchical chain of translational evidence [432]. Collectively, CIBP models provide a reproducible platform for target validation and intervention assessment, whilst underscoring the necessity for finer stratification and reporting regarding cell line selection, host immune background, temporal phases, and outcome measures to enhance clinical translatability [433].

The complete Freund's adjuvant (CFA)‐induced inflammation model and the carrageenan‐induced inflammation model are pivotal animal models for investigating chronic inflammatory pain, simulating persistent pain states arising from tissue injury and inflammation. In these models, the local inflammatory response at the injury site triggers the release of a cascade of algesic mediators, including prostaglandins, cytokines, and kinins, which collectively act upon primary sensory neurons to induce nociceptive sensitization. For instance, intraplantar injection of carrageenan in rats induces marked inflammation and hyperalgesia; research has identified that various inflammatory mediators play critical roles in nociceptor sensitization during this process [434, 435]. Through investigations utilizing these models, scientists have identified numerous cellular and molecular targets involved in inflammatory pain, forming the basis for the development of anti‐inflammatory analgesic drugs or therapies [415]. However, inflammatory pain models possess limitations: a single acute inflammatory stimulus is difficult to fully recapitulate the complex immune–neuro interactions present in human chronic pain states. Furthermore, these models primarily assess evoked pain responses following stimulation, which remains distinct from the continuous spontaneous pain experienced by patients [436].

Animal models have played an indispensable role in elucidating chronic pain mechanisms and screening novel pharmacotherapeutics. Through appropriate models, researchers can deeply dissect the genesis of distinct pain types and preliminarily validate the efficacy and safety of novel analgesic candidates [414]. However, it must be emphasized that significant differences exist between animals and humans, and models cannot comprehensively simulate all facets of chronic pain. For instance, the CCI model primarily reflects peripheral nerve injury pain, inadequately capturing the complexity of pain resulting from central nervous system injury; moreover, the degree of nerve compression induced by CCI surgery can vary across experiments, leading to substantial variability in results [415]. More broadly, physiological differences exist between the nervous systems of rodents (mice and rats) and humans. Pain assessment in animals relies heavily on somatic reflexes or avoidance responses, failing to embody the subjective affective dimensions of the human pain experience. These discrepancies contribute to the failure of many analgesic strategies effective in animal models to reproduce expected efficacy in clinical trials [437]. Consequently, current research efforts are directed toward both refining traditional models and developing novel ones—such as transgenic pain models or primate models that more closely approximate human pathology—to enhance clinical relevance and predictability [433]. In summary, while preclinical animal models provide invaluable insights for chronic pain research, effectively translating animal experimental findings to the clinic remains a formidable challenge.

### 5.2 Innovative Therapies in Clinical Trials

Despite the availability of numerous pharmacological agents, the clinical management of chronic pain remains suboptimal, often hampered by systemic toxicities and modest efficacy. The current analgesic landscape is undergoing a fundamental shift from broad‐spectrum symptom suppression toward a phenotype‐based and mechanism‐driven paradigm. This evolution is fueled by a deeper understanding of the distinct molecular drivers underlying neuropathic, inflammatory, and nociplastic pain, as well as the integration of nonpharmacological modalities that target neural circuits directly.

To navigate this complex landscape, this section categorizes emerging therapies into four primary pillars: ion channel‐targeting agents, neuro–immune and metabolic modulators, gene and cell therapies, and digital or bioelectronic medicine. By addressing the specific maladaptive plasticity of the nervous system, these strategies aim to overcome the inherent limitations of standard‐of‐care treatments—such as the CNS‐related side effects of gabapentinoids or the cardiovascular risks of NSAIDs—offering a more precise and durable therapeutic window.

#### 5.2.1 Ion Channel‐Targeting Therapies: Selective Nav1.7/1.8 Inhibitors

Voltage‐gated sodium channels Nav1.7 and Nav1.8 play pivotal roles in regulating nociceptor excitability and are regarded as novel targets for nonopioid analgesia [438, 439, 440]. Among them, Nav1.7 has garnered significant attention due to its prominence in human pain genetics. Given that Nav1.7 is primarily expressed in peripheral primary sensory neurons, relatively selective blockade holds the promise of potent analgesia without compromising central nervous system function. Numerous pharmaceutical companies and research institutions have invested in the development of selective Nav1.7 inhibitors, achieving encouraging analgesic effects in rodent pain models. However, to date, no Nav1.7 inhibitor has succeeded in Phase II clinical trials: multiple candidates failed to demonstrate superiority over placebo in patients [436]. This discrepancy exposes significant gaps between preclinical and clinical testing, prompting deep reflection on cross‐species translational issues. Analyses reveal that, taking Nav1.7 inhibitors as an example, many preclinical studies are predominantly conducted on young male rodents [441], utilizing inflammatory pain models and focusing on evoked pain responses. In contrast, clinical trials often target populations with complex chronic neuropathic pain characterized by spontaneous pain requiring long‐term medication. Furthermore, animal experiments frequently employ single‐dose acute administration, whereas clinical settings necessitate repeated dosing to observe efficacy; these design differences may contribute to inconsistent outcomes.

By comparison, Nav1.8 is another sensory neuron‐specific sodium channel, primarily mediating action potential conduction in small‐diameter neurons. Selective inhibitors targeting Nav1.8 have recently shown promise in clinical trials for acute pain: for instance, the oral small molecule suzetrigine (VX‐548) significantly reduced pain intensity in postoperative patients, with the high‐dose group meeting the primary analgesic endpoint [442]. In early 2025, the US FDA approved suzetrigine for the treatment of moderate‐to‐severe acute pain, marking it as the first analgesic with a novel mechanism of action approved in decades and raising hopes for a breakthrough in opioid‐free analgesia [443]. Although Nav1.8 inhibitors have primarily demonstrated efficacy in acute pain, their role in chronic pain requires further investigation; nonetheless, this progress undoubtedly bolsters confidence in ion channel‐targeted analgesic strategies. Collectively, therapies targeting peripheral sensory nerve ion channels offer the potential for high selectivity and fewer side effects, a feasibility repeatedly demonstrated in preclinical studies [444]. The key to future success lies in optimizing clinical trial designs, selecting appropriate patient populations, and utilizing relevant evaluation metrics to fully validate the true efficacy of such therapies.

#### 5.2.2 Neuro–Immune Targeting Therapies: Glial Inhibitors and Cytokine Antagonists

Chronic pain involves not only alterations in neuronal excitability but also critical neuro–immune interactions that maintain and amplify nociception. Injury or sustained stimulation can trigger the activation of central and peripheral glial cells, releasing a plethora of proinflammatory factors that further enhance sensory neuron excitability [415]. Consequently, therapies modulating neuro–immune responses have emerged as a novel direction for chronic pain treatment. In preclinical studies, broad‐spectrum agents inhibiting glial activation have demonstrated analgesic effects. For instance, the antibiotic minocycline, which suppresses microglial activation and proinflammatory signaling pathways, attenuated hyperalgesia across various nerve injury pain models and reduced the expression of pain‐related molecules such as BDNF in the spinal cord [445]. A randomized controlled trial in patients with lumbar radicular pain compared oral minocycline with amitriptyline; results showed some pain relief in both groups over a 2‐week treatment period, with fewer side effects in the minocycline group, although the overall magnitude of analgesia was modest, necessitating validation in larger, longer‐term trials [446]. Another study administered prophylactic minocycline to patients undergoing spinal surgery to prevent postoperative chronic pain but found no significant difference overall, suggesting potential efficacy only in a subgroup of patients with severe spontaneous pain [447]. These findings imply that the clinical analgesic efficacy of glial inhibitors may depend on factors such as timing of administration and patient pain phenotype, warranting further exploration [448, 449, 450]. Beyond antibiotics, novel drugs specifically targeting glial pathways are under development. For example, the P2×7 receptor, an ATP‐gated ion channel on the microglial surface, induces the release of cytokines like IL‐1β upon activation [451]. P2×7 antagonists have been shown to alleviate neuropathic pain and suppress neuroinflammation in animal models, with candidates currently in clinical trials evaluating safety and efficacy [452, 453, 454]. Similarly, blockers targeting another crucial glial signaling pathway—the fractalkine (CX3CL1)/CX3CR1 axis, a glial‐derived inflammatory factor—hold promise for inhibiting neuron–glia crosstalk to relieve pain, with efficacy currently being investigated in animal studies [455, 456, 457].

Concurrently, proinflammatory cytokine antagonists have demonstrated potential in certain refractory pain states. As previously mentioned, inflammatory mediators such as TNF‐α and IL‐1β play significant roles in the maintenance of chronic pain [415]. Biologics targeting these mediators, originally intended for inflammatory diseases like rheumatoid arthritis, are now being trialed for pain management. For instance, the IL‐1 receptor antagonist anakinra was recently utilized in a clinical trial for refractory CRPS. In this study, patients with long‐standing severe CRPS received daily subcutaneous injections of anakinra for 120 days (interleukin‐1 receptor antagonist treatment for refractory CRPS (v1)). Results indicated that mean pain intensity decreased from a baseline of 7.7/10 to 5.3/10 at the end of treatment, representing an approximate one‐third reduction. Most patients experienced improvements in limb function, mood, and QoL during treatment; 14 patients showed a marked reduction in pain scores, with only a minority showing no change or worsening symptoms. Anakinra was generally well tolerated, with no serious adverse events reported. At the 2‐month follow‐up after discontinuation, pain rebounded to some extent but remained improved compared with baseline. Researchers concluded that IL‐1 antagonism demonstrates efficacy and feasibility in CRPS, warranting larger‐scale placebo‐controlled trials [211, 212, 213, 458]. Beyond IL‐1, antitumor necrosis factor (anti‐TNF) agents have been explored in clinical studies for radicular pain and OA pain; some reports suggest that local application of anti‐TNF may help alleviate sciatica or knee OA pain, though evidence remains inconsistent [459]. Overall, therapies targeting neuro–immune pathways (glial inhibition and cytokine antagonism) offer novel perspectives for intractable chronic pain. Preclinical evidence strongly supports the association between these mechanisms and pain [415]. The current challenge lies in translating these findings into safe and effective human therapies, necessitating meticulously designed clinical trials to determine optimal delivery sites, therapeutic windows, and target populations to fully realize their analgesic potential.

#### 5.2.3 Gene and Cell Therapies

With advancements in genetic engineering and cell transplantation technologies, researchers are attempting to fundamentally remodel molecular pathways associated with pain, aiming to achieve sustained analgesia. regarding gene therapy, the delivery of therapeutic genes to the nervous system via nonviral or viral vectors to modulate pain‐related gene expression represents a frontier of exploration [460, 461]. Classical approaches include using nonreplicating HSV‐1 viral vectors to introduce the preproenkephalin (*PENK*) gene into sensory neurons, enabling local release of endogenous opioid peptides at the site of pain to alleviate symptoms [462, 463]. In animal models, HSV vectors carrying the *PENK* gene have significantly attenuated pain behaviors in models of inflammatory pain [464], neuropathic pain [462, 465], and even bone cancer pain [466]. Based on these findings, researchers conducted the first human trial of gene therapy for pain: injecting an HSV vector encoding the PENK gene near the ganglia of patients with cancer pain. Phase I clinical results demonstrated safety and feasibility, with some patients achieving relief from intractable cancer pain, laying the groundwork for further investigation [467]. Beyond endorphins, HSV vectors have also been utilized to deliver GABA synthesis enzyme genes to enhance inhibitory neurotransmitter production, or anti‐inflammatory cytokine genes to counteract pain‐associated inflammatory responses, all yielding positive outcomes in animal studies [468, 469, 470].

Another novel avenue involves directly targeting and regulating the gene expression of key molecules in nociceptive signal transduction. For instance, the aforementioned phenomenon where Nav1.7 gene mutations lead to insensitivity to pain has inspired gene therapy strategies. A research team utilized CRISPR/dCas9 fused with a transcriptional repressor domain to construct an epigenetic silencing tool specifically targeting the Nav1.7 gene, delivering it to murine DRG via adeno‐associated virus vectors [471]. In models of inflammatory pain and chemotherapy‐induced neuropathy, a single intrathecal injection of this gene therapy significantly elevated pain thresholds and reduced hyperalgesia, with durable efficacy: effects persisted for at least 44 weeks in the inflammatory pain model and 15 weeks in the chemotherapy‐induced neuropathy model. Crucially, this therapy did not induce complete sensory loss or motor dysfunction; mice exhibited a “tuned down” pain sensitivity without numbness or behavioral abnormalities. The study also replicated these analgesic effects using zinc finger protein (ZFP)‐mediated Nav1.7 gene silencing. As dCas9 is bacterial in origin and may elicit immune responses, researchers posit that human protein‐based ZFPs may be more clinically viable. This work establishes a foundation for gene therapy in chronic pain: by nonpermanently downregulating the expression of key genes in nociceptive pathways, long‐acting analgesia can be achieved while avoiding the risks associated with permanent pain blockade. Currently, this platform technology is being advanced to the clinical translation stage by spin‐off companies.

Regarding cell therapy, the transplantation of stem cells and their derivatives is viewed as a regenerative medicine approach with the potential to cure intractable pain. Most notable is the application of mesenchymal stem cells (MSCs) in chronic pain. MSCs possess immunomodulatory and proreparative properties, capable of secreting various growth factors, anti‐inflammatory cytokines, and exosomes to remodel the microenvironment of damaged tissues. Given that chronic pain pathology often involves local neuroinflammation, tissue degeneration, or ischemia, MSC transplantation holds the potential to simultaneously intervene in these pathological processes, thereby achieving both pain relief and the promotion of disease reversal [472]. In animal studies, MSCs have demonstrated efficacy in reducing neuroinflammation, promoting nerve regeneration, and attenuating pain behaviors in neuropathic pain models. In clinical studies for conditions such as OA, intra‐articular injection of MSCs has been reported to reduce pain and improve joint function over a defined period [473, 474]. For refractory CRPS, the US National Institutes of Health recently funded a pioneering study intending to utilize human MSCs to treat the neuro–immune abnormalities involved in CRPS. This project, awarded a $5.5 million grant, aims to manufacture high‐quality clinical‐grade MSCs and determine optimal dosing regimens, with plans to launch the first human trial within 2 years. If successful, it would become the first mechanism‐based therapy for CRPS, potentially not only alleviating pain but also modifying the disease course. The project leader noted that the core of CRPS lies in neuro–immune dysregulation, and MSCs possess multiple mechanisms of action precisely suited to correct this imbalance, including the secretion of anti‐inflammatory factors and the promotion of tissue regeneration and immune tolerance. Furthermore, the application of MSCs in other diseases has established a safety profile, suggesting that with appropriate methodologies, they could be rapidly advanced to the clinic. Beyond MSCs, researchers are also exploring the transplantation of iPSC‐derived neural progenitor cells into the spinal cord to replace and repair damaged interneuron pathways, or utilizing engineered immune cells to enter the nervous system and suppress aberrant inflammatory responses. These cell therapies are currently mostly in animal experimentation or early clinical stages, and their efficacy and long‐term safety require further validation. Nevertheless, it is foreseeable that with a deeper understanding of chronic pain biology, cell and gene therapies will offer novel means to intervene in pain mechanisms at their source. Once technologies mature, they have the potential to provide revolutionary treatment options for certain intractable pain conditions.

#### 5.2.4 Digital Therapeutics

Beyond pharmacological and biotechnological modalities, digital therapeutics grounded in internet and wearable technologies have emerged prominently in pain management in recent years. Digital therapeutics refer to clinically validated software or hardware programs that improve clinical outcomes through specific interventions. For chronic pain patients, digital therapeutics focus particularly on behavioral and neuromodulatory interventions, including virtual reality (VR) therapy and application (App)‐based CBT programmes.

VR technology, through immersive audiovisual environments, distracts patient attention from pain and integrates interactive behavioral training, serving as an adjunctive treatment for acute and chronic pain. Studies indicate that VR can effectively alleviate acute pain sensations such as postoperative pain and burn dressing change pain, whilst also improving mood and sleep in chronic pain patients [475]. A VR program developed specifically for CLBP patients (RelieVRx, formerly EaseVRx) comprises an 8‐week home‐based VR training course incorporating CBT techniques such as breathing relaxation, attention diversion, and pain reconceptualization. This therapy significantly reduced pain intensity and distress in randomized controlled trials and received de novo authorization from the US FDA in November 2021, becoming the world's first VR digital therapeutic approved for chronic pain management [476]. RelieVRx is approved as a prescription digital therapeutic to be used alongside conventional treatment for CLBP to reduce pain and its interference with daily life. This milestone establishes a new status for VR analgesia in the medical field and paves the way for regulatory approval of subsequent VR therapies. The advantages of VR therapy lie in its noninvasiveness and reusability. In clinical application, patients simply wear a dedicated headset to undergo guided immersive training at home, deriving analgesic benefits without medication. A recent large‐scale community trial showed that after 56 home VR sessions, patients' mean pain intensity decreased by approximately 2.0 points from baseline (on a 0–10 scale), and pain interference with daily life dropped by 2.3 points, with effects persisting for at least 3 months after cessation of training [477]. These data further consolidate the clinical value of VR digital therapeutics. Nevertheless, VR therapy faces challenges, such as temporary simulator sickness in a small subset of patients (approximately 5% reported mild nausea, which resolved rapidly). Future efforts should refine the usability and personalization of VR content to enhance patient adherence and the durability of efficacy.

CBT stands as a core nonpharmacological intervention for chronic pain, aiming to alleviate suffering and improve function by altering patients' cognition and coping behaviors regarding pain. Traditional CBT requires face‐to‐face or telephonic guidance from trained therapists, which is time consuming and resource intensive. Leveraging mobile internet technology, self‐guided or AI‐assisted CBT applications have emerged, enabling patients to access continuous psychological and behavioral guidance via smartphones. A series of studies and trials demonstrate that the efficacy of such digital CBT interventions for chronic pain is comparable to that of therapist‐delivered treatments. A multicenter randomized controlled trial published in 2022 compared AI‐guided mobile CBT with therapist‐delivered telephonic CBT in patients with chronic back pain. The results indicated that after 3 months of intervention, the magnitude of improvement in pain‐related disability scores was equivalent between the two groups, with the mobile CBT regimen proving noninferior to the therapist‐led protocol [478]. Crucially, AI‐driven CBT achieved equivalent efficacy with only approximately half the therapist time investment. This implies that delivering CBT via intelligent applications holds the potential to substantially expand access to high‐quality psychological interventions across the population, serving more patients in need with limited therapist resources. Currently, numerous CBT/mindfulness training apps targeting chronic pain are available on the market, with some achieving positive results in clinical trials. For instance, the “Curable” app, which combines education, writing exercises, and meditation, has been proven to significantly reduce self‐reported intensity of various chronic pain conditions after 6 weeks of use; the “PainCoach” app improved patients' physical function and QoL through personalized daily exercises [479]. Another related digital therapeutic is digital ACT; for example, a 12‐week mobile ACT program developed for fibromyalgia patients demonstrated more pronounced pain relief and mood improvement than the control group in a randomized trial. These studies corroborate the feasibility of app‐based behavioral therapies. With the advancement of artificial intelligence, chatbot technology has also been introduced into the domain of psychological intervention for pain. For instance, conversational AI agents such as “Wysa” can engage in text‐based communication with users, offering emotional support and guidance on CBT techniques; preliminary studies indicate that they are effective in alleviating psychological distress [480, 481]. Overall, digital therapeutics are demonstrating an increasingly pivotal role in chronic pain management. They are capable of transcending temporal and geographical constraints, delivering evidence‐based interventions to a broad patient population in a low‐cost and highly accessible manner. Concurrently, digital platforms facilitate the collection of vast amounts of real‐time data, which can be utilized to personalize treatment regimens and assist in clinical decision‐making. For example, by monitoring patient activity and sleep via wearable devices and integrating these data with pain diaries within apps, clinicians can gain a more comprehensive understanding of the patient's status and dynamically optimize therapy.

The translation from animal models to clinical trials constitutes a critical bottleneck in contemporary pain research and drug development; nevertheless, the advancements in the emerging therapies discussed above undoubtedly offer renewed hope for patients with chronic pain. From a preclinical perspective, despite the inherent limitations of animal models, they have been instrumental in elucidating the multifarious mechanisms of pain genesis and modulation, thereby delineating directions for subsequent therapeutic development [415]. Currently, a spectrum of therapies grounded in novel mechanisms—ranging from pharmacological agents targeting ion channels and neuro–immune pathways to frontier biotechnologies such as gene editing and cell transplantation, as well as digital health interventions—are progressively bridging the chasm from “concept to clinic.” Although some candidates have failed to meet expectations in early trials, exemplified by the challenge of insufficient clinical efficacy encountered by Nav1.7 inhibitors [436], others have exhibited the dawn of success, such as the breakthrough of Nav1.8 inhibitors for postoperative pain and the regulatory approval of VR therapies. It is foreseeable that the future of chronic pain management will be characterized by multidisciplinary convergence, where pharmacotherapy, gene/cell therapies, and digital therapeutics act synergistically to deliver mechanism‐based treatments tailored to individual pathological profiles. To realize this objective, a more robust translational research ecosystem is required—one that fully accounts for the complex characteristics of human pain at the preclinical stage and enhances the signal detection and success rates of early clinical studies through innovative trial designs. In summary, emerging therapies present an opportunity for a paradigm shift in the treatment of chronic pain. As reviewed in this section, both novel biomedical strategies targeting nociceptive signaling pathways and digital interventions addressing patients' psychological and lifestyle factors demonstrate immense potential. Through sustained research and multicenter clinical validation, we anticipate the advent of a more effective and comprehensive chronic pain treatment architecture, enabling patients to truly benefit from the promise of a “pain‐free” future driven by scientific progress [411].

#### 5.2.5 Synthesis of Clinical Benchmarks and Translational Evidence

The translation of novel analgesic concepts from preclinical models to bedside efficacy is encapsulated in the following reference tables, which collectively illustrate the paradigm shift toward precision pain medicine.

Table 3 provides a clinical management overview, directly contrasting established first‐line standards—spanning traditional antidepressants, gabapentinoids, and anti‐inflammatory agents—with emerging therapeutic frontiers. This comparison highlights how novel modalities like selective NaV blockers and gene therapies aim to achieve peripheral precision while avoiding the dose‐limiting sedation and systemic toxicities often associated with conventional pharmacological management. Furthermore, it underscores the strategic shift toward addressing nociplastic pain through digital therapeutics and neuromodulation, which target centralized maladaptive connectivity rather than peripheral tissue damage alone.

**TABLE 3:** Comparative management strategies: current standards versus emerging frontiers by pain type.

Pain phenotype	Standard therapy	Mechanism and limitations	Emerging therapies	Mechanism and potential advantages	References
Neuropathic pain	Gabapentinoids (pregabalin, gabapentin) Antidepressants (duloxetine, amitriptyline) Topical agents (lidocaine, capsaicin)	Mech: CaVα2δ subunit blockade; Enhance descending inhibition. Limit: systemic sedation, dizziness, weight gain; moderate efficacy.	Selective Nav blockers (Nav1.7/1.8 inhibitors) Gene therapy (CRISPR‐Nav1.7, HSV‐enkephalin) Neuromodulation (high‐freq SCS, DRG‐S)	Precision targeting of peripheral nociceptors without CNS sedation “One‐and‐done” long‐term silencing; reversal of maladaptive plasticity Paresthesia‐free analgesia; focal coverage	[122, 123, 124, 315, 327, 330, 331, 394, 462, 463]
Inflammatory/nociceptive Pain	NSAIDs/COX‐2 inhibitors Acetaminophen Corticosteroid injections	Mech: prostaglandin inhibition; anti‐inflammatory. Limit: GI bleeding, CV risk, renal toxicity; cartilage degradation (steroids)	Anti‐NGF antibodies (tanezumab) Resolvins/specialized proresolving mediators Bioelectronic medicine (vagus nerve stimulation)	Blockade of key sensitization driver (NGF); high potency Active promotion of inflammation resolution vs. mere suppression Activation of cholinergic anti‐inflammatory pathway; nonpharmacological	[266, 267, 268, 305, 306, 344, 380]
xed/Nociplastic Pain	Exercise/CBT SNRIs (duloxetine) Interdisciplinary rehab	Mech: restore function; modulate central processing Limit: high patient effort required; adherence barriers; limited biological specificity	Digital therapeutics (VR, App‐based CBT) Psychedelic‐assisted therapy Brain stimulation (tDCS, TMS)	Accessible, scalable behavioral modification; rewiring of fear‐avoidance circuits Rapid “reset” of DMN connectivity Direct modulation of cortical excitability	[75, 76, 316, 317, 318, 356, 357, 358, 359, 360, 361, 362, 363, 364, 365, 366, 367, 368]

Complementing this framework, Table 4 details the translational status of representative mechanism‐based therapies. These data reflect a dynamic pipeline where advanced candidates such as NaV1.8 inhibitors and basivertebral nerve ablation have demonstrated robust clinical signals, while earlier frontiers targeting neuro–immune axes or neurotrophin modulation represent high‐potential future directions. Together, these summaries delineate a transition toward “disease‐modifying” analgesia designed to resolve underlying pathophysiology and reset the nervous system's functional architecture rather than merely suppressing symptoms.

**TABLE 4:** Translational landscape of mechanism‐based analgesic therapies: from preclinical evidence to clinical trials.

Pathway and mechanism of action	Preclinical evidence and animal models	Drug (sponsor)	Target indication	NCT No./phase/status	Objectives	Preliminary findings	References
NaV1.8 selective inhibition: blocks voltage‐gated sodium channel 1.8 on peripheral nociceptors to reduce pain transmission without central opioid‐like effects	Demonstrated in CCI (chronic constriction injury), SNL (spinal nerve ligation), and CFA models: reverses mechanical allodynia and thermal hyperalgesia by suppressing peripheral nociceptor excitability	Suzetrigine/VX‐548 (vertex)	Painful diabetic peripheral neuropathy (DPN)	NCT06696443/Phase 3/active, not recruiting	Evaluate long‐term safety, tolerability, and long‐term effectiveness in DPN pain	NCT06696443: ongoing; no results posted. Phase 2 DPN: Week‐12 mean NPRS change −2.26/−2.11/−2.18 (high/mid/low), pregabalin reference −2.09; generally well tolerated; no study‐drug‐related SAEs	[414, 415]
NaV1.8 selective inhibition: blocks peripheral sensory neuron excitability to reduce nociceptive signaling	Evaluated in postincisional and neuropathic models (CCI/SNL): effectively reduces action potential firing in DRG neurons and mitigates acute/chronic pain behaviors	LTG‐001 (Latigo Biotherapeutics)	Acute postoperative pain after third molar extraction	NCT06774625/Phase 2/completed	Evaluate efficacy and safety of LTG‐001 for acute pain after impacted third molar removal	Phase 2: completed; no results posted publicly. Phase 1 (healthy volunteers, *n* = 72): well tolerated; *T*_max_ ≈1.5 h; predictable PK; US FDA Fast Track granted	[418, 419]
Neurotrophin modulation (NT‐3): p75NTR‐Fc fusion protein to scavenge excess neurotrophins and modulate sensitization in osteoarthritis pain	Tested in surgical DMM (destabilization of medial meniscus) and MIA‐induced OA models: preclinical studies suggest that targeting neurotrophin signaling reduces nociception in OA models, although effects on joint structural progression remain incompletely defined	LEVI‐04 (Levicept)	Knee osteoarthritis pain	NCT05618782/Phase 2a/completed	Assess efficacy and safety of repeated IV dosing vs. placebo over 20 weeks	Phase 2 RCT: significant improvements vs. placebo across doses at Weeks 5 and 17; >50% achieved ≥50% pain reduction and >25% achieved ≥75% at Weeks 5 and 17; no increased incidence of RPOA or joint pathologies vs. placebo	[482, 483]
LPA1 receptor antagonism (CNS‐penetrant): targets lysophosphatidic acid signaling implicated in neuroinflammation and pain processing	Validated in SNL and neuroinflammation models: Blocks LPA‐induced demyelination, glial activation, and effectively reverses neuropathic mechanical allodynia	PIPE‐791 (Contineum Therapeutics)	Chronic osteoarthritis pain (COAP)/chronic low back pain (CLBP)	NCT06810245/Phase 1/Active, not recruiting	Assess safety/tolerability; explore effects on pain over crossover periods	NCT06810245: no results posted. Phase 1b PET: high brain receptor occupancy with PK–target engagement correlation	[192, 484, 485]
Dual NOP/MOP receptor agonism: engages nociceptin (NOP) and μ‐opioid (MOP) receptors to provide strong analgesia with potentially improved tolerability	Confirmed in SNL, CCI, and acute incisional models: Produces potent, broad‐spectrum analgesia with a wider therapeutic window and fewer opioid‐related side effects	Cebranopadol (Tris Pharma)	Moderate‐to‐severe acute postoperative pain (registrational); chronic pain development planned	NCT06545097/Phase 3/completed	Evaluate analgesic efficacy and safety vs. placebo after abdominoplasty (AUC4‐48)	Topline (ALLEVIATE‐1): met primary endpoint; AUC4‐48 LS mean difference 59.2 (SE 14.36) vs. placebo; *p* < 0.001; generally well tolerated; no cebranopadol‐related SAEs	[420, 421, 486]
Endocannabinoid system modulation: THC‐focused nano modulator delivered transmucosally as add‐on therapy	Observed in CCI, SNL, and CIBP (cancer‐induced bone pain) models: CB1/CB2 activation suppresses spinal nociceptive transmission and modulates neuro–immune interactions	AP707 (Apurano Pharmaceuticals)	Chronic pain due to central neuropathy	NCT06071949/Phase 2/recruiting	Assess changes in pain intensity, sleep, and quality of life over 14 weeks	Trial ongoing; no publicly posted outcomes identified in registry at time of access	[487, 488]
Inflammasome‐related neuroinflammation: proposed NLRP3/NEK7‐axis inhibition to reduce inflammatory pain signaling	Demonstrated in CFA and carrageenan‐induced inflammatory models: reduces the release of the “inflammatory soup” (IL‐1β, IL‐6), strongly mitigating peripheral sensitization	HT‐6184 (Halia Therapeutics)	Postprocedural pain/inflammation after third molar extraction	NCT06241742/Phase 2/completed	Test ability to attenuate biomarkers of inflammation and reduce pain postprocedure	Completed; ClinicalTrials.gov record indicates no results posted.	[435, 443]
PACAP ligand blockade (neuropeptide signaling): inhibits PACAP‐mediated migraine biology (chronic pain spectrum)	Supported by nitroglycerin‐induced and trigeminal sensitization models: blocks PACAP38‐induced dural vasodilation, trigeminal nociceptive activation, and photophobia	Lu AG09222 (Lundbeck)	Migraine prevention in patients with prior preventive failures	NCT05133323/Phase 2a/completed	Reduce monthly migraine days over Weeks 1–4 vs. placebo after single IV infusion	HOPE trial (reported): baseline 16.7 migraine days/month; Weeks 1–4 change −6.2 days (750 mg) vs. −4.2 days placebo; difference −2.0 days (95% CI −3.8 to −0.3); *p* = 0.02	[489, 490, 491, 492]
Central neuromodulation (M1 rTMS): modulates pain networks/descending inhibition via motor cortex stimulation	Evaluated in reserpine‐induced centralized pain and CCI models: modulates maladaptive neuroplasticity and enhances descending inhibitory pathways in motor–thalamic–cortical loops	Motor cortex rTMS (procedure)	Fibromyalgia	NCT03658694/completed	Evaluate add‐on M1 rTMS vs. sham; primary outcome ≥50% pain reduction at Week 8	International multicenter sham‐controlled trial: 99.4% Bayesian probability of ≥50% pain reduction at Week 8 with active rTMS vs. sham (OR 3.04; NNT 4.54); effects waned by Week 16	[493, 494]
Spinal cord stimulation (10 kHz): high‐frequency paresthesia‐free neuromodulation of neuropathic pain circuits	Investigated in STZ‐induced diabetic neuropathy and SNL models: high‐frequency stimulation alters dorsal horn wide‐dynamic‐range (WDR) neuron hyperexcitability and neuron‐glia crosstalk without paresthesia	10 kHz SCS + conventional medical management (Nevro)	Refractory painful diabetic neuropathy (PDN)	SENZA‐PDN (ClinicalTrials.gov: NCT03228420)/RCT follow‐up	Assess durability of pain relief and functional/neurologic outcomes	24‐month results: mean pain reduction 79.9% vs. preimplantation; 90.1% responders (≥50% pain relief; 128/142); ∼66% improved neurological status at 24 months	[422, 495]
Glia/immune modulation hypothesis: low‐dose naltrexone (opioid receptor antagonist; proposed TLR4/glia effects)	Validated in CCI, SNL, and acidic saline‐induced centralized pain models: TLR4 antagonism on microglia attenuates proinflammatory cytokine release and reverses central sensitization	Naltrexone 6 mg (trial drug)	Fibromyalgia	NCT04270877/RCT/completed	Test whether 12‐week LDN is superior to placebo for pain reduction	Randomized trial: mean pain change −1.3 vs. −0.9; between‐group difference −0.34 (95% CI −0.95 to 0.27); *p* = 0.27; similar AE rates; no deaths	[496, 497, 498]

*Data sources*: ClinicalTrials.gov records for NCT06696443, NCT06774625, NCT05618782, NCT06810245, NCT06545097, NCT06071949, NCT06241742, and NCT05133323 (accessed on March 31, 2026).

## 6 Discussion

This review has systematically delineated epidemiological, pathophysiological, and management strategies landscapes of chronic pain. A coherent consensus emerges: chronic pain is not a unitary entity but rather a heterogeneous complex of syndromes driven by the multilevel interplay of genetic, epigenetic, neural, immune, metabolic, and psychosocial factors. Its central pathological characteristic is “maladaptive plasticity” of the nervous system—a plasticity that manifests across peripheral, spinal, cerebral, and systemic levels, ultimately consolidating into a pathological state that is refractory to reversal.

Although our understanding of ion channels, glial cells, neuro–immune crosstalk, central circuit remodeling, and even epigenetic “memory” has reached unprecedented depths, as detailed in Section 3, and innovative modalities such as interventional procedures, neuromodulation, gene/cell therapies, and digital therapeutics are emerging, as illustrated in Sections 4 and 5, a stark reality remains: a formidable “translational chasm” persists between profound mechanistic insights and substantial clinical benefit. The root of this chasm lies in our continued inability to effectively deconstruct the immense heterogeneity of chronic pain.

### 6.1 Critical Bottlenecks: Individual Heterogeneity, Biomarker Paucity, and the Failure of Translational Models

The primary challenge facing contemporary pain research and management is the precise alignment of “mechanism” with “patient.” First, there is a profound scarcity of clinical biomarkers for mechanistic stratification. As discussed in Section 3.6, while modalities such as TSPO–PET, fMRI, QST, and IENFD show promise, no single or combined biomarker is currently used routinely in clinical practice to mechanistically stratify chronic pain patients. We remain unable to reliably determine clinically whether a patient's pain is predominantly driven by peripheral sensitization, central sensitization, neuroinflammation, or autoimmunity. This “black box” status precipitates the dilemma discussed in Section 5.2.1: despite immense investment in Nav1.7 research within animal models, clinical trials have repeatedly failed. This failure likely stems not from target invalidity, but from the inability to precisely recruit the subgroup of patients with “Nav1.7‐driven” pain. Second, the limitations of preclinical models and the obscurity of “chronification” mechanisms. The animal models summarized in Section 5.1, such as CCI, SNL, and CIBP, are effective in simulating specific pain phenotypes but suffer from inherent deficiencies in recapitulating key dimensions of human chronic pain: they are largely evoked pain models, struggling to capture spontaneous pain and affective–cognitive dimensions; they predominantly utilize young male animals, neglecting sexual dimorphism and ageing effects; and they fail to adequately model the “transition switch” from acute to chronic states—the process of “pain chronification.” A major gap in current research is elucidating why, following similar acute injuries, some individuals recover while others develop chronic pain. This likely involves the interplay of acute‐phase inflammatory intensity, early epigenetic “programming,” and psychosocial factors, yet the critical nodes and modifiable “temporal windows” remain unclear. Finally, the dilemma of integrating the BPS model. While the BPS model is universally accepted, in practice, biological research and psychosocial research are often bifurcated. We know that stress (HPA axis) and the microbiome (gut–brain axis) influence pain, but exactly how do they modulate spinal microglial activation or cerebral predictive coding circuits? Conversely, how do psychological/digital interventions like CBT or VR remodel functional brain connectivity or even peripheral immune status? Current management paradigms tend toward “stacking” rather than “integration”—patients may receive medication, physiotherapy, and psychological counseling concurrently, but these interventions are not synergistically designed based on a unified mechanistic understanding.

Furthermore, fundamental biological divergences between species contribute significantly to the attrition of mechanism‐based therapies. A paradigmatic example is the failure of glial inhibitors in clinical settings. While minocycline and P2×4 antagonists robustly reverse neuropathic pain in rodent models by suppressing microglial activation, their efficacy in humans has been equivocal or negligible [443, 444]. This discrepancy may stem from distinct genomic and functional profiles of human versus murine microglia; for instance, key signaling pathways like TLR4 exhibit species‐specific ligand recognition properties, rendering targets validated in mice potentially irrelevant in human pathology [499]. Moreover, animal models typically rely on evoked reflexive withdrawal thresholds as a proxy for pain, which poorly correlates with the spontaneous, affective, and fluctuating nature of human chronic pain. Consequently, candidates that successfully normalize reflex thresholds in homogenous, young, male animal cohorts often fail to alleviate the complex, subjective suffering experienced by heterogeneous patient populations.

### 6.2 Future Directions: From “Symptom Management” to “Mechanism Modification”

To bridge the aforementioned chasm, future research must pivot from “describing mechanisms” to “deconstructing heterogeneity,” and from “empirical treatment” to “mechanism‐driven precision intervention.” First, the core breakthrough lies in developing and validating a panel of biomarkers capable of reflecting individual pathophysiological states. This necessitates longitudinal, large‐scale cohort studies integrating multiomics (genomics, epigenomics, transcriptomics, metabolomics, microbiome), deep phenotyping (QST, digital biomarkers), and multimodal imaging (fMRI, PET). The objective is to transcend current coarse classifications based on etiology or anatomy and achieve stratification based on dominant mechanistic “endophenotypes,” such as classifying patients into “ion channel type,” “neuroinflammation type,” “central inhibition deficit type,” or “autoimmune type.” This will serve as the cornerstone for precision pharmacotherapy. Second, research focus should shift from “established” chronic pain to the “nascent” acute‐to‐chronic transition phase. Utilizing human iPSC models and more refined animal models, combined with longitudinal patient tracking, we must identify early molecular and circuit signals of “chronification.” This offers the potential for developing “disease‐modifying drugs” (DMDs)—interventions applied briefly during the acute phase to “reset” neural plasticity and prevent the consolidation of pain memory. Finally, disciplinary barriers must be dismantled. Interventions capable of simultaneously modulating central circuits and peripheral inflammation should be developed. More importantly, the synergy between different therapies must be understood. For instance, can CBT or mindfulness practice enhance descending inhibition, thereby augmenting the efficacy of SCS? Can daily activity and mood data collected via digital therapeutics serve as biomarkers to guide medication titration? Such a “closed‐loop” management model, fusing psychological intervention (CBT), digital phenotyping (app data), and biomedical treatment (SCS/drugs), represents the requisite path toward true integration of the BPS model.

## 7 Conclusion

Chronic pain stands as one of the most formidable medical challenges of the 21st century [410, 500]. It is not merely a symptom but a complex chronic disease entity involving multisystem dysregulation [501]. This review has systematically elucidated our contemporary understanding of this ancient affliction across multiple dimensions, encompassing epidemiology, pathophysiological mechanisms, clinical management strategies, and frontier therapies. It is evident that over the past two decades, pain science has undergone a paradigmatic revolution, shifting from a “peripheral‐centric” view to a multidimensional network perspective encompassing “central–immune–genetic–psychological” interactions [502]. Our comprehension of nociceptive signaling [503], sensitization maintenance [504], and central remodeling [505] has penetrated to molecular and circuit levels. However, the brilliance of these fundamental research achievements contrasts sharply with the relative lag in clinical management. The crux of this disparity lies in the translational gap created by “heterogeneity” [506]. Current clinical practice remains tethered to coarse classifications based on symptom description and etiology, with treatment relying predominantly on trial‐and‐error elimination [507]. To achieve a genuine breakthrough in chronic pain management, a transition is imperative: moving from “one‐size‐fits‐all” symptomatic treatment to precision intervention grounded in individualized pathological mechanisms.

Looking forward, the field of chronic pain stands at a crossroads. The path ahead demands close collaboration among basic researchers, clinicians, engineers, and patients. As our understanding of heterogeneity deepens and technological breakthroughs emerge, there is reason to believe that in the near future, we will achieve a fundamental transformation from “controlling pain” to “curing pain.”

## Author Contributions

Z.S., Z.T., and Z.W. performed the comprehensive literature search and drafted the original manuscript. S.X., X.L., and G.T. conceived the topic, designed the structure of the review, and provided supervision. Z.Y., Y.Y., Z.W., and Z.W. were responsible for the visualization and creation of the figures. Z.W., Y.G., and X.H. prepared the tables and assisted with reference management and formatting. All authors critically reviewed the manuscript and approved the final version for submission.

## Funding

This work was supported by National Key R&D Program (2025YFC3508900, 2025YFC3508901), National Natural Science Foundation of China (81973623, 82574753), National High Level Traditional Chinese Medicine Hospital Clinical Research Funding (DZMG‐LJRC0011), Jiangsu Provincial Scientific Research Program on Traditional Chinese Medicine and Integrated Traditional Chinese and Western Medicine (CYTF2026114), and 2025 Danyang Municipal Competitive Projects of the Special Fund for Scientific and Technological Innovation (Social Development) (SSF202516).

## Ethics Statement

The authors have nothing to report.

## Conflicts of Interest

The authors declare no conflicts of interest.

## Data Availability

The authors have nothing to report.
